# Towards efficient near-infrared fluorescent organic light-emitting diodes

**DOI:** 10.1038/s41377-020-00456-8

**Published:** 2021-01-21

**Authors:** Alessandro Minotto, Ibrahim Bulut, Alexandros G. Rapidis, Giuseppe Carnicella, Maddalena Patrini, Eugenio Lunedei, Harry L. Anderson, Franco Cacialli

**Affiliations:** 1 Department of Physics and Astronomy and London Centre for Nanotechnology, University College London, London, WC1E 6BT UK; 2 Department of Chemistry, Chemistry Research Laboratory, University of Oxford, Mansfield Road, Oxford, OX1 3TA UK; 3 Dipartimento di Fisica, Università degli Studi di Pavia, Via A. Bassi, 6, Pavia, 27100 Italy; 4 CNR-ISMN, Istituto per lo Studio dei Materiali Nanostrutturati, Consiglio Nazionale delle Ricerche, Via P. Gobetti 101, Bologna, 40129 Italy

**Keywords:** Organic LEDs, Polymers, Green photonics

## Abstract

The energy gap law (*E*_G_-law) and aggregation quenching are the main limitations to overcome in the design of near-infrared (NIR) organic emitters. Here, we achieve unprecedented results by synergistically addressing both of these limitations. First, we propose porphyrin oligomers with increasing length to attenuate the effects of the *E*_G_ -law by suppressing the non-radiative rate growth, and to increase the radiative rate via enhancement of the oscillator strength. Second, we design side chains to suppress aggregation quenching. We find that the logarithmic rate of variation in the non-radiative rate vs. *E*_G_ is suppressed by an order of magnitude with respect to previous studies, and we complement this breakthrough by demonstrating organic light-emitting diodes with an average external quantum efficiency of ~1.1%, which is very promising for a heavy-metal-free 850 nm emitter. We also present a novel quantitative model of the internal quantum efficiency for active layers supporting triplet-to-singlet conversion. These results provide a general strategy for designing high-luminance NIR emitters.

## Light-emitting diodes: Near-infrared fluorescence from organic molecules

Organic (carbon-based) light-emitting diodes (LEDs) that emit near-infrared light can be built by linking together large organic molecules called porphyrins, offering many potential industrial and medical applications. Organic near-infrared LEDs have several advantages over conventional LEDs based on inorganic semiconductors, including mechanical flexibility, biocompatibility and the absence of polluting heavy metals. Researchers in the UK and Italy led by Harry Anderson at the University of Oxford and Franco Cacialli at University College London explored the potential of linked porphyrin structures that fluoresce at near-infrared wavelengths. The optical properties of the materials are improved by engineering the molecular structure and a quantitative model is presented to explain the efficient emission. This research provides understanding of exciton dynamics and points towards innovative uses of near-infrared light in applications including light therapy, optical communications, biosensors and biometric systems.

## Introduction

Near-infrared (NIR) emitters are attracting significant interest for integration in a variety of applications, spanning from photodynamic therapy^1^ to security and defense^2^. Since NIR radiation (here defined as wavelengths 700 nm < *λ* < 1000 nm) is mostly invisible to the human eye, NIR light-emitting diodes (LEDs) are also among the best candidates for the development of Li-Fi (light-fidelity) all-optical networking systems, which offer a promising approach to the alleviation of bandwidth limitations often already affecting wireless (Wi-Fi) systems^3,4^. Furthermore, with respect to inorganic emitters, organic NIR emitters offer the possibility to further extend the range of applications thanks to their mechanical flexibility, conformability, and biocompatibility.

Most of the recent research on NIR organic light-emitting diodes (OLEDs)^5^ has focused on rare-earth and transition metal complexes^6,7^, small molecules^8,9^, conjugated polymers, and their combinations^9–14^. However, the emission efficiency of organic emitters in the NIR is hindered by some intrinsic limitations. First, the extended conjugation length needed to achieve a sufficiently small energy gap (*E*_G_) dictates a very planar molecular conformation, which in turn favors the formation of poorly emissive H-type aggregates^15^. Undesired intermolecular interactions can be suppressed in conjugated systems by diluting the chromophores in solid solutions^13,14,16–19^ or via molecular design^20^, including threading into cyclodextrin rings to form conjugated polyrotaxanes^21–23^.

A potentially more significant hurdle is represented by the so-called “energy-gap law” (*E*_G_-law) for radiationless transitions^7,24^, which predicts that the rate of the (non-radiative) transition between two electronic states increases exponentially as the energy difference (*E*_G_) between the states is decreased. In fact, a concomitant decrease in the photoluminescence yield with *E*_G_ is commonly observed experimentally^25^, although it is difficult, in general, to disentangle this contribution from that of aggregation.

Hybrid organic/inorganic innovative materials such as perovskite methylammonium lead halides^26^ and quantum dots^27^ may offer an alternative with high external quantum efficiency (EQE), but their heavy metal content will eventually prevent their use in most applications, especially biocompatible or wearable ones. Toxicity issues can also affect phosphorescent materials incorporating toxic heavy elements^6,26,28–32^.

Emission in the NIR by using heavy-metal-free materials has been achieved, for example, by leveraging triplets (e.g., via reverse intersystem crossing (RISC), or so-called “thermally activated delayed fluorescence (TADF)”) but so far only at wavelengths <800 nm for high efficiency devices^9,33,34^ and, interestingly, also with fluorescent porphyrin oligomers^18^, whose conjugation length can be extended essentially across the entire molecule if the porphyrins are connected via conjugated bridges featuring one (*meso-*ethyne links^35–37^) or two triple bonds (*meso*-butadiyne links^18,38^). This scheme is particularly appealing for its potential tuneability, as by increasing the number of porphyrin units, the emission can be tuned from the visible range to wavelengths above 850 nm^39^, while still maintaining photoluminescence (PL) efficiencies of approximately 30% in dilute solution^40^.

## Results

**Fig. 1:**
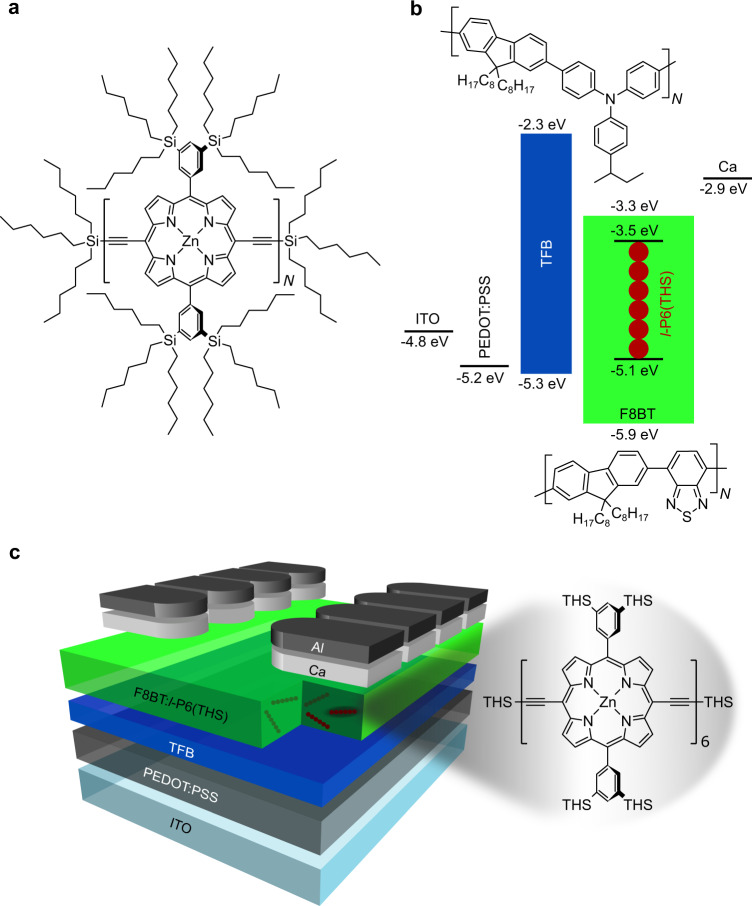
Molecular structure of l-PN(THS) oligomers and OLED illustration. **a** Molecular structure of the l-PN(THS) oligomer series. **b** Band diagram for the materials employed in the OLEDs^18,58^. TFB (poly[(9,9-dioctylfluorenyl-2,7-diyl)-alt-(4,4′-(N-(4-sec-butylphenyl)diphenylamine)]) and F8BT molecular structures are illustrated above and below the relative band diagrams, respectively. **c** OLED architecture including an ITO patterned glass substrate, a poly(3,4-ethylene dioxythiophene) doped with poly(styrene sulfonate) (PEDOT:PSS) hole-transport layer, a TFB electron/exciton blocking layer, an F8BT:l-P6(THS) NIR light-emitting layer and a Ca/Al cathode

Here, we report the optical properties of a series of linear *meso*-butadiyne-linked zinc porphyrin oligomers (*l-*P*N*(THS) Fig. 1), and demonstrate unprecedentedly high EL EQEs by blending the zinc porphyrin hexamer *l-*P6(THS) in poly[(9,9-di-*n*-octylfluorenyl-2,7-diyl)-*alt-*(benzo[2,1,3]thiadiazol-4,8-diyl)] (F8BT). The insertion of closed-shell zinc(II) metal ions into these porphyrin chains does not have a strong effect on their photophysical behavior, and the corresponding magnesium(II) complexes or free-base oligomers are expected to have similar properties. The choice of F8BT as a polymer host was driven mainly by its excellent semiconducting properties and energy transfer considerations; although we also note that the singlet exciton formation yield in F8BT OLEDs has been found to exceed the spin statistical limit of 25% in previous reports^41,42^.

In terms of emitter engineering, first, we propose a series of fluorescent heavy-metal-free porphyrin oligomers with increasing length (1–6 repeat units). Conjugated triple-bond-based bridges between the porphyrins allow effective intramolecular electronic coupling among the macrocycles, and thus enable the singlet exciton to delocalize over increasing portions of the molecule, thereby forcing an increasing mismatch of the spatial extent of the singlet and of the triplet excitons in view of the intrinsically localized nature of the triplets. Such a mismatch is expected to suppress intersystem crossing (ISC) and therefore the nonradiative rate (*k*_nr_). In addition, exciton delocalisation is also expected to favour decoupling from vibrational ladders^7^. In fact, the growth of the nonradiative rate as a function of the decrease in *E*_G_ is characterized in our systems by a logarithmic rate approximately an order of magnitude smaller than in previous studies^43–45^. Moreover, the effective intramolecular electronic coupling among the porphyrins allows us to boost oscillator strength and thus radiative rate with increasing length. Second, we added trihexylsilyl (THS) side-chains to the aryl groups of the porphyrins to prevent aggregation quenching more effectively than in a previously reported linear hexamer^18^ thanks to steric hindrance by the THS groups, which limits π–π interactions.

We are able to complement the above breakthrough by demonstrating EL peaked at a remarkably long wavelength of 850 nm from OLEDs with a polymeric active layer in which the hexamer is blended. Such OLEDs exhibited an average maximum EQE of 1.1%, with values of up to 3.8% for the best performing devices. Such efficiencies are the highest reported so far for a heavy-metal-free NIR fluorescent emitter in this spectral region (>800 nm).

We also present a quantitative model that we have developed to describe the quantum efficiency of devices incorporating a guest–host emitter blend in which the host is susceptible to triplet–triplet annihilation (TTA) or TADF to afford further insight into device physics. The model supports the presence of TADF in addition to TTA as a mechanism to generate singlets via triplet conversion, whose existence is in turn supported by time-resolved EL data.

### Optical properties of l-PN(THS) oligomers in solution

**Fig. 2:**
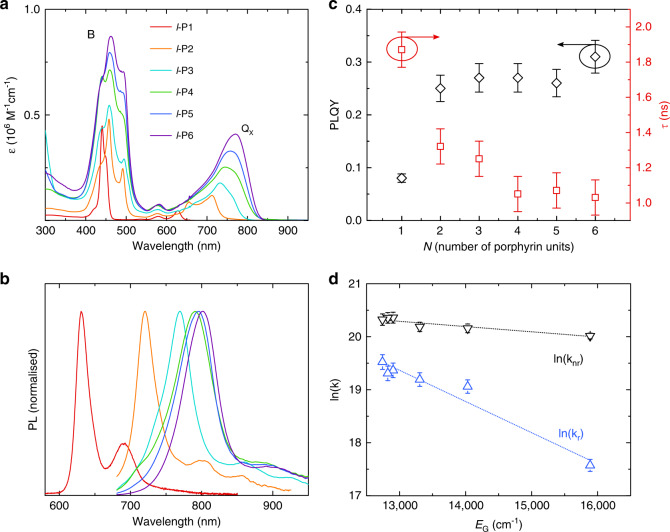
Solution optical properties of l-PN(THS) oligomers. **a** Molar absorption coefficient (*ε*) of the l-PN(THS) oligomer series in toluene solution. **b** PL spectra of the l-PN(THS) oligomers in toluene solution (~1 μM) measured at room temperature following excitation at 450 nm. **c** Experimental PL quantum yield (PLQY) (black diamonds) and PL lifetime (red squares) versus oligomer length measured in ~1 μM toluene solutions at room temperature following excitation at 450 nm (PLQY) and 405 nm (PL lifetime). **d** Natural logarithm of radiative (*k*_r_, blue triangles) and non-radiative (*k*_nr_, black triangles) rates versus energy gap (*E*_G_). The values of *k*_r_ and *k*_nr_ (in s^−1^ units) were derived from the experimental PLQY and PL lifetimes. The absolute value of the slope of ln(*k*_nr_) is ~0.86 ± 0.16 eV^−1^, i.e., more than one order of magnitude lower than those obtained from different series of fluorescent and phosphorescent dyes in previous studies (∼10 eV^−1^)^43–45^. Dotted lines represent linear fits of the ln(*k*_r_) and ln(*k*_nr_) data. The linear fit of ln(*k*_r_) is presented only as a guide to the eye to highlight the different trend with *N* (decreasing *E*_G_) compared to ln(*k*_nr_).

The absorption and PL spectra of the *l-*P*N*(THS) oligomers, measured in diluted toluene solution (~10^−6^ M), are illustrated in Fig. 2.

The main spectral components in both the absorption and PL spectra are common to all the oligomers in the series and resemble those of similar non-THS-substituted oligomers^38–40^. The high-energy absorption band (“Soret-band”) is due to transitions to the higher excited state (*S*_0_ − *S*_n_) and peaks in the region 450–550 nm^38^, whereas the low-energy band (“Q-band”, *S*_0_ − *S*_1_ transition) is a manifold of species dominated by a low-energy component^38^. This component, named *Q*_X_, is strongly influenced by the number of porphyrin units in the oligomer.

As shown in Fig. 2a, the oscillator strength of the *Q*_X_ transition increases with the number of porphyrin units due to the alignment of the corresponding dipole moment along the main axis of the oligomer, and the extended conjugation across the entire oligomer length^46^. For the same reasons, with increasing oligomer size, the *Q*_X_-band also gradually red-shifts from 630 nm (*l*-P1(THS)) to 770 nm (*l*-P6(THS)). The overall broadening of the Q-band with increasing oligomer length can be ascribed to the torsional heterogeneity of the porphyrin units along the main axis, enabled by the *meso*-butadiyne links^47,48^.

In contrast to the ground state, in the *S*_1_ excited state, from which fluorescence arises, the torsional disorder is reduced thanks to the much steeper potential energy surface, which favors a planar conformation. For this reason, twisted conformers that are excited from *S*_0_ to *S*_1_ tend to planarize prior to emission (on the ~100 ps timescale)^39,47,48^. As a consequence, the fluorescence spectra in solution (Fig. 2b) exhibit a relatively narrow (70–130 meV full width at half maximum, FWHM) peak, which shifts from 630 nm to 800 nm with the number of porphyrins, and secondary vibronic components in the lower energy tail of the spectrum. An important property for NIR applications is that the fraction of photons emitted in the NIR (>700 nm) increases from 13% (*l*-P1(THS)) for the monomer to >90% for the dimer, reaching 100% for the hexamer.

In Fig. 2c, we present the PL efficiency values for the *l-*P*N*(THS) series in toluene solution and the corresponding PL lifetimes (*τ*), which we obtained by fitting the mono-exponential PL decay of the same solutions (see Fig. S1, Supplementary Information). As expected, the lifetimes are ~1–2 ns, confirming the singlet nature of the radiative exciton, and decrease monotonically from ~1.9 ns for *l*-P1 to ~1.0 ns for *N* > 3. This decrease is due to the increase in both the radiative (*k*_r_) and non-radiative rates (*k*_nr_) with oligomer length, as discussed below.

Most notably, however, as shown in Fig. 2c, the PLQY for *N* > 2 essentially saturates at ~28 ± 3%, more than three times higher than the PLQY of the monomer (8%); this is rather surprising considering the decrease in the energy gap, i.e., this result is in apparent contrast with the predictions of the *E*_G_ law.

In fact, from the trends in the experimentally determined rates (Fig. 2d) extracted by combining the experimental PLQY and *τ* (Fig. 2c), we see that *k*_nr_ is not increasing nearly as dramatically as would have been expected on the basis of previous experiments^43–45^, contrary to the expectation of efficient internal conversion from *S*_1_ to *S*_0_. We propose that the increase in *k*_nr_ is prevented by the concomitant suppression of ISC, which is expected to decrease significantly with increasing oligomer length, mainly as a result of the increasing difference in spatial extent between singlets and triplets^46,49,50^, and possibly by suppression of exciton-vibrations coupling^7^.

Figure 2d provides additional insight into the advantage of our material design strategy in affording a high PLQY by the simultaneous manipulation of both radiative and non-radiative processes. Specifically, we highlight the dependence of *k*_r_ on *E*_G_ as a consequence of the increasing oscillator strength with *N*.

Overall, both the enhancement of *k*_r_ and the suppression of ISC/excitonic-vibrational coupling in *l-*P*N*(THS) oligomers result in an exception to the corollary of the *E*_G_-law, i.e., that the PLQY should decrease with decreasing *E*_G_. This is very encouraging in the context of NIR emitters, as it allows us to develop devices with unprecedented EQEs by incorporating them into appropriate host matrices.

### Optical properties of *l*-P6(THS) in polymer matrices

To take advantage of these promising properties and prevent aggregation quenching in solid-state devices, the next hurdle is the identification of a host featuring good charge transport and optical properties, especially in regard to spectral overlap and thus resonant energy transfer. Here, we selected the polyfluorene derivatives F8BT and poly(9,9-dioctylfluorene-*alt*-*N*-(4-*sec*-butylphenyl)-diphenylamine) (TFB), which gave the best results (F8BT) for a number of low-gap emitters in previous work^14,18,19^, or should have yielded good spectral overlap (TFB) between the host emission and the Soret bands of the porphyrins (the strongest ones, Fig. 2). As an emitter, we chose the hexamer among all oligomers because of its highest PLQY and NIR spectral purity.

**Fig. 3:**
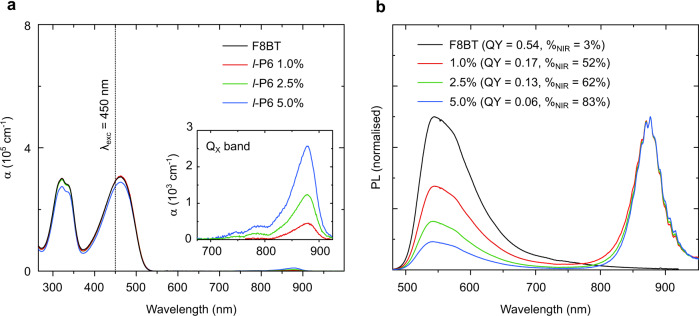
Optical properties of F8BT:l-P6(THS) blends in solid-state thin films. **a** Absorption spectra of F8BT:l-P6(THS) blend thin films at different hexamer concentrations and **b** relative PL spectra normalized with respect to the intensity of emission at 875 nm (at 545 nm for the neat F8BT film). The inset in **a** highlights the evolution of the *Q*_X_ l-P6(THS) absorption with increasing hexamer concentration. **b** The relative PLQY values (measured over the whole emission spectrum) are reported in the legend, together with the fraction of NIR emission (%_NIR_)

The absorption and PL spectra for pure F8BT and blends with *l*-P6(THS) are reported in Fig. 3. The results for the TFB blends, which, despite the better spectral overlap, yielded devices with poorer performance, are reported in Figs. S3 and S4 and Tables S2 and S3 (Supplementary Information). We attribute the better performance of F8BT-based devices to better transport properties and higher singlet exciton formation rates (i.e., leveraging triplet-triplet annihilation or thermally activated delayed fluorescence, *vide infra*)^41,42^.

As illustrated in Fig. 3a, the UV/Vis part of the blend film absorption spectrum is dominated by the F8BT components at 325 and 465 nm, whereas the *Q*_X_ band of *l*-P6(THS) rises in the 800–900 nm range, with a maximum varying from 850 to 875 nm (inset in Fig. 3a).

Most notably, however, we observe a significant red-shift (~75 nm) of the *Q*_X_ band of *l*-P6(THS) in the F8BT matrix compared to the spectrum measured for the solution (Fig. 2a and Fig. S5 in the Supplementary Information section). In addition, as highlighted in the inset of Fig. 3a, the *Q*_X_ band is 50 nm narrower than in solution and exhibits some secondary components at higher energy, which are not merely the consequence of a well-defined vibronic progression. Instead, both the substantial red-shift and spectral redistribution of the oscillator strength can be attributed to a higher planarity, and reduced torsional heterogeneity of the hexamer in the solid state (either in a neat film or diluted in an F8BT matrix, Fig. S5 in the Supplementary Information section) compared to the solution, in which each porphyrin unit is free to rotate around the butadiyne links with a continuous range of torsion angles^47^. As previously reported^48^, when the torsional degrees of freedom are constrained, for instance, when the oligomer is in a solvent with high viscosity or embedded in a solid-state matrix, planar and twisted conformers correspond to distinct spectroscopic species, each exhibiting characteristic absorption features in the *Q*_X_ manifold. Incidentally, the *Q*_X_ band profile of the blended films resembles the one we measured from a neat *l*-P6(THS) film (Fig. S5a, Supplementary Information), although in the latter case the contribution to the absorption from twisted conformers is better resolved, likely as a result of unhindered packing interactions in the absence of a solid matrix.

In the PL spectra presented in Fig. 3b, we note that emission from F8BT is progressively quenched in favor of emission from *l*-P6(THS) with increasing hexamer concentration, thanks to the host–guest resonant energy transfer afforded by the spectral overlap between the host emission and the high-energy components of the hexamer Q-band. Similar to the absorption, the PL from the solid-state films is considerably red-shifted compared to that in solution and exhibits a similar scattering of the maximum value depending on the sample position investigated. We attribute this red-shift to a combination of solvation effects and planarization of the porphyrin hexamer in the solid blends. In addition, we do not observe any secondary emission at higher energy, originating from twisted conformers, as previously reported from PL measurements carried out on similar oligomers in toluene solution^48^. The absence of such high-energy PL components can also be reasonably ascribed to the close packing of the hexamers in the solid state, which favors exciton funnelling towards the lowest-emitting planar conformers. Additionally, the rigidity of the oligomers in the solid-state matrix might also be responsible for the minimum Stokes’ shift (<10 nm) on all samples, analogous to what was observed in porphyrin oligomer complexes^39^ and ladder-like conjugated polymers such as poly(*para*-phenylene)^51^. However, this minimal Stokes’ shift, together with the red-shift in the absorption (and PL) spectrum, may also be related to either intraoligomer, oligomer–oligomer, or polymer–oligomer dipole coupling in a J-aggregated manner^52^.

Concerning the PL efficiency, we summarized in the legend of Fig. 3b and in Table S1 the PLQY values of films with up to 10 w/w% hexamer loading. Notably, these values exceed 10% in the solid state without the need for additives to limit the formation of poorly emissive aggregates^18^. This achievement represents a radical advance compared to the linear P6 hexamer previously reported^18^, for which the PLQY of the F8BT:P6 blend increased from <1 to 11% only after the addition of the metal coordinating 4-benzylpyridine.

From the different blends we tested, we note that the samples with 1 and 2.5 w/w% *l*-P6(THS)/polymer concentrations gave the best trade-off between high PLQY and the % of NIR emitted photons (Fig. 3). Therefore, we focused on these two concentrations for testing as active layers in OLEDs.

### OLEDs

**Fig. 4:**
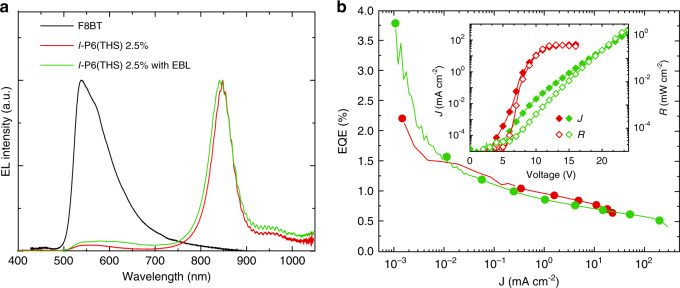
OLED characteristics. EL spectra of the OLEDs incorporating F8BT:l-P6(THS) as the active layer collected at 15 and 24 V (i.e., the maximum radiance voltages) without and with EBL, respectively. **a** EQE versus current density (**b**), and corresponding JVR curves (inset)

The breakthrough above was confirmed by the fabrication and characterization of F8BT:*l*-P6(THS)-based OLEDs, with the device structure illustrated in Fig. 1c. The devices that gave the best NIR performance were those based on the blend with 2.5 w/w% *l*-P6(THS) loading. The spectra, current–voltage–radiance (JVR) and EQE versus current density characteristics are plotted in Fig. 4. The main performance parameters are summarized in Table 1.

**Table 1:** Summary of the OLED performance parameters

Sample	*V*_ON_^a^ [V]	<*R*_MAX_> ^b^ [mW cm^−2^]	EQE_MAX_^c^ [%]	<EQE_MAX_> ^d^ [%]	EL in NIR^e^ [%]
F8BT:*l*-P6(THS) 2.5%	5.6 ± 1.2	0.3 ± 0.1	2.2	0.88 ± 0.35	98
F8BT:*l*-P6(THS) 2.5% with EBL^f^	6.7 ± 0.9	1.9 ± 0.6	3.8	1.10 ± 0.50	95

^a^Voltage at which the light output exceeds the noise level, as extrapolated from the radiance-voltage characteristics.

^b^Average maximum radiance (for 16 devices).

^c^Maximum external quantum efficiency.

^d^Average external quantum efficiency.

^e^Photons emitted in the near-infrared spectral region (i.e., *λ* > 700 nm).

^f^Exciton/electron blocking layer.

These OLEDs were characterized by spectrally pure NIR EL up to the highest voltages tested here (Fig. 4a), in this particular case with >98% (and more generally >95% over the set of all devices we tested) of the photons emitted in the NIR region (at 15 V) and an EL maximum at 850 nm. Quenching of the F8BT emission in EL was significantly higher than that observed in photoluminescence (in which only 62% of the photons are emitted from the *l*-P6(THS) dopant), thereby giving evidence for the direct formation of a significant quota of the emissive excitons on the porphyrins. EL from F8BT was also observed to increase slightly with applied voltage (see Fig. S6 in the Supplementary Information for a different diode, for which the F8BT contribution to emission is slightly higher but never higher than 5% at the highest voltage). An additional contribution to the spectral purity is a full width at half maximum (FWHM) of only ~60 nm, which is very narrow for this spectral range compared to previously reported NIR organic emitters^6,8,10,12,14^. Conjugated luminophores generally exhibit broad emission (and absorption) bands in the NIR region, in most cases because of conformational disorder, the charge-transfer nature of the emitting species, or both. The distinctively narrow emission band of our devices is desirable for spectroscopy (especially with a view to bio-applications and diagnostics), as it enables spectral selectivity.

Moving then to examine the performance of F8BT:*l*-P6(THS) OLEDs, we observe that devices without the EBL exhibited a turn-on voltage (*V*_ON_) of ~5 V and a maximum radiance of 0.4 mW/cm^2^ (Table 1). Such devices exhibited a 2.2% maximum EQE: remarkably high for OLEDs with heavy-metal-free active layers emitting at 850 nm. Although we measured this maximum value at a current density of 0.001 mA/cm^2^, the average maximum EQE (i.e., the average of the maximum measured over each device, for 16 diodes) was more than respectable at 0.88 ± 0.35% and remained above 0.6% for higher current densities (up to 30 mA/cm^2^).

With the two-fold aim of suppressing exciton quenching at the interface with poly(3,4-ethylene dioxythiophene) doped with poly(styrene sulfonate) (PEDOT:PSS) and improving electron-hole balance, we also fabricated diodes incorporating an additional thin (<10 nm) TFB exciton/electron blocking layer (EBL)^53,54^ between the PEDOT:PSS injection layer and the emissive layer (Fig. 1c). As shown in Fig. 4b, despite the presence of a slightly larger proportion of F8BT emission (but at higher applied voltage, see Fig. 4a, *vide infra*) compared to the devices without the EBL, we obtained significantly higher (five times) average maximum radiances (1.9 mW/cm^2^ Table 1). Most strikingly, however, we increased the average maximum EQE up to 1.1%, with the best performing device yielding a maximum EQE (EQE_MAX_) = 3.8%. Although the devices were still affected by some roll-off of the efficiency with increasing current, in comparison to previously reported THS-free linear porphyrin hexamers (P6) with no benzylpyridine, the maximum EQE was two orders of magnitude higher^18^. We note that a “roll-off” of the efficiency with increasing current also occurred in other host-blend systems and characterized early phosphorescent OLEDs in particular but has now mostly been addressed by appropriate device and materials engineering, and we expect similar advances to be possible for the systems we present here. Therefore, the presence of such roll-off does not affect the fundamental importance of our findings regarding the general strategy to enhance luminescence in this particularly challenging spectral window.

A more detailed comparison between *l*-P6(THS) and *l*-P6(THS-free) OLEDs is reported in the Supplementary Information section (Fig. S7 and Table S4) together with results on neat F8BT control devices, that are less efficient than the NIR-emitting devices (thus ruling out that the high efficiency of the NIR OLEDs might be due to F8BT emission, please see Supplementary Information section 11 for a full discussion).

### A quantitative electroluminescence model

We next consider the ratio of singlets to the total number of formed excitons (*r*_st_), and note that the derivation of the latter on the basis of the maximum EQEs reported in Table 1, and by using the usual expression of the IQE as the product of the electron-hole-balance factor (*γ*) of *r*_st_ and of the PL efficiency *η* is likely to be inaccurate, as it would ignore any triplet-to-singlet conversion process. In fact, the values derived in this way (50 ± 5% and 30 ± 3% in OLEDs with and without EBL, respectively, see section 13 of the Supplementary Information section) apparently exceed the spin-statistics limit (25%) even for conservative estimates of *γ* (*γ* = 1) and of the outcoupling factor (*ξ* = 0.75/*n*^2^), i.e., assuming all emitting chromophores oriented parallel to the device plane, with *n* obtained via ellypsometry in our case—see Fig. S8 in the Supplementary Information section. Notably, triplet-to-singlet conversion mechanisms such as TTA and RISC/TADF have indeed been shown to increase the apparent *r*_st_ over the 25% limit, not only for rubrene-doped systems^55^ but also for (neat) F8BT diodes in particular^41,42^. This is clearly relevant to our devices for which F8BT is the host.

To obtain more accurate estimates of *r*_st_, we thus developed a new quantitative model of the EQE, that not only considers singlet generation from triplets via either TTA or TADF but also, crucially, considers exciton generation directly on the guest, alongside energy transfer from the host to the guest. More specifically, in this model, the EL efficiency of the guest (porphyrin hexamer in our case) is written as the sum of two contributions: the first contribution is due to the radiative decay of singlets formed in F8BT and then transferred resonantly to *l*-P6(THS), and the second contribution is from singlets directly formed at the hexamer site. The details of such a model for the EQE of blended devices are reported in Supplementary Information section 13 and yield an *r*_st_ of ~63 and ~67% under the conservative assumptions of perfect charge balance (*γ* = 1) and an outcoupling efficiency *ξ* ~ 0.24 (i.e., 0.75/*n*^2^ for in-plane dipole orientation)^42^. In principle, if the emission could be forced to happen at an (unrealistically sharp) ideal distance from the reflective electrode (*ξ* ~ 1.2/*n*^2^ ~0.38), in addition to assuming an overestimated quantum yield of the porphyrin hexamers (taken to be the same as in the diluted solution, ~0.31, thus disregarding any solid-state quenching that is known to happen also in low-concentration solid solutions) and perfect electron-hole balance (*γ* = 1), the lower limit for $r_{{\rm{stP}}6}^ \ast$ (defined as the “effective” singlet to total number of excitons ratio, i.e. including the contribution from singlets generated in F8BT via TTA or thermally-activated RISC) would be 0.4 for the best performing device without EBL and 0.42 for the one with EBL. However, these assumptions would be overconservative, and we do not think they would appropriately capture the device physics (e.g., the spatial distribution of the emission region). Taken together, our results thus point to a singlet population including well over 40% of the total excitons (the value derived by Wallikewitz et al.^41^, as the sum of the 25% initially generated singlets and 15% additional singlets generated via a chain of consecutive TTA reactions only), thus implying a significantly greater contribution of RISC in addition to or in place of TTA.

## Discussion

In conclusion, we have investigated a series of fluorescent heavy-metal-free porphyrin oligomers with increasing length to increase the mismatch of the spatial extent of singlets and triplets in order to reduce ISC, and thus mitigate the increase in the non-radiative rate *k*_nr_ while concomitantly increasing the oscillator strength (and radiative rate, *k*_r_). We have also combined this strategy with the molecular design of side chains to suppress aggregation quenching, thereby achieving PL efficiencies upto 30% and emission at wavelengths well above 800 nm.

The basic photophysics and material design breakthrough has been confirmed by incorporating an F8BT:l-P6(THS) blend into OLEDs, with which we demonstrated an average EQE of 1.1% and a maximum EQE of 3.8% at a peak wavelength of 850 nm. We analysed the results within the frame of a novel quantitative model, which implies the importance of RISC/TADF to account for our EQE values beyond the apparent limit imposed by spin statistics. The EQEs presented here are, to the best of our knowledge, the highest reported so far in this spectral range from a heavy-metal-free fluorescent emitter.

Not only do our results demonstrate milder increases in *k*_nr_ with (decreasing) *E*_G_ than in the literature, but most importantly, they also provide a general strategy for designing high-luminance NIR emitters. In the short term, they may enable the further development of OLEDs in this challenging spectral range for a wide range of potential applications spanning the life sciences (biochemical wearable sensors, in vivo sub-surface bio-imaging, to name just two), security (e.g., biometrics), horticulture, and (in)visible light communications (iVLC), a serious contestant to alleviate the bandwidth demands of the imminent Internet-of-Things (IoT) revolution. Most importantly, in perspective, these findings are significant to a range of disciplines.

## Materials and methods

### Materials and thin-film PL characterization

The synthesis of the *l*-P6(THS) oligomer has been previously reported^56^. Thin films were spin-cast onto fused silica substrates from a 10 mg/mL toluene solution of the F8BT:*l*-P6(THS) blends. The host polymers (F8BT and TFB) were purchased from America Dye Source. The films were deposited in a N_2_ environment via spin-coating at 2000 rpm for the F8BT blends and 1000 rpm for the TFB blends to obtain a thickness of ~100 nm, measured with a Dektak profilometer. Photoluminescence was collected from an Andor Shamrock 163 spectrograph coupled with an Andor Newton EMCCD. The PLQY characterization was carried out using a 450 nm diode laser (Thorlabs) and an integrating sphere setup (Bentham)^57^. All absorption and PL spectra were collected in air at room temperature.

### Transient PL characterization

Time-resolved PL measurements were carried out in air at room temperature with a time-correlated single photon counting (TCSPC) spectrometer (LifeSpec Edinburgh Instruments). Samples were excited at 405 and 445 nm with picosecond pulsed diode lasers.

### OLED fabrication and characterization

ITO substrates were cleaned in an ultrasonic bath using acetone and isopropanol, dried under a N_2_ stream and treated in an O_2_ plasma chamber for 10 min. A 40 nm layer of PEDOT:PSS (purchased from Sigma Aldrich) was spin-coated at 4000 rpm from a 2.8 wt% dispersion in water. The active layer was spin-coated from the same solutions used for the PL characterization, and a Ca/Al (30/200 nm) cathode was thermally evaporated on top. For the multilayered OLEDs, a film of TFB was spin-cast onto PEDOT:PSS from a 10 mg/ml solution in toluene at 3000 rpm and then annealed at ~185 °C for 1 h in a N_2_ environment. The TFB film was then spin-rinsed with toluene to obtain a final thickness of <10 nm^53^, measured with a Dimension Icon atomic force microscope. The samples were then measured under an ~10^−2^ mbar vacuum using a Keithley 2400 source-meter for both the current measurement and the voltage supply. The optical output of the OLEDs was measured with a calibrated silicon photodiode, and the EL spectra were collected with the same spectrometer employed for the PL experiments.

## Supplementary information

Supplementary Information-Towards efficient near-infrared fluorescent organic light-emitting diodes
