# Supplementary material for: Towards efficient near-infrared fluorescent organic light-emitting diodes
Source: Light Sci Appl. 2021 Jan 21;10:18. doi: 10.1038/s41377-020-00456-8 (PMC7820436; doi:10.1038/s41377-020-00456-8)
Supplement: Supplementary file 1 — Supplementary Information-Towards efficient near-infrared fluorescent organic light-emitting diodes [file 41377_2020_456_MOESM1_ESM.docx]

**Supplementary Information**

**Towards efficient near-infrared fluorescent organic light-emitting diodes**

Alessandro Minotto^1^, Ibrahim Bulut^2^, Alexandros G. Rapidis^1^, Giuseppe Carnicella^1^, Maddalena Patrini^3^, Eugenio Lunedei^4^, Harry L. Anderson^2^*, and Franco Cacialli^1^*

^1^Department of Physics and Astronomy and London Centre for Nanotechnology

University College London, London WC1E 6BT, United Kingdom

^2^Department of Chemistry, University of Oxford, Chemistry Research Laboratory, Mansfield Road, Oxford OX1 3TA, United Kingdom

^3^Dipartimento di Fisica, Università degli Studi di Pavia, Via A. Bassi, 6, Pavia 27100, Italy

^4^CNR-ISMN, Istituto per lo Studio dei Materiali Nanostrutturati, Consiglio Nazionale delle Ricerche, Via P. Gobetti 101, Bologna 40129, Italy

*email: [harry.anderson@chem.ox.ac.uk](mailto:harry.anderson@chem.ox.ac.uk), [f.cacialli@ucl.ac.uk](mailto:f.cacialli@ucl.ac.uk)

**Contents**

[1. Experimental Section 3](#_Toc60681728)

[2. Transient photoluminescence of the *l*-P*N* oligomers in solution 4](#_Toc60681729)

[3. Radiative rate calculation 5](#_Toc60681730)

[4. PLQY of the F8BT:*l*-P6(THS) and TFB: *l*-P6(THS) solid-state blends 8](#_Toc60681731)

[5. Absorption and PL spectra of the TFB:*l*-P6(THS) solid-state blends 9](#_Toc60681732)

[6. TFB:*l*-P6(THS) OLEDs 10](#_Toc60681733)

[7. Absorption and PL of spectra of neat *l*-P6(THS) films 12](#_Toc60681734)

[8. Applied voltage dependence of EL spectra 13](#_Toc60681735)

[9. F8BT:*l*-P6(THS-free) hexamer PLEDs 14](#_Toc60681736)

[10. Refractive indices via spectroscopic ellipsometry 17](#_Toc60681737)

[11. Neat F8BT PLEDs and efficiency roll-off 18](#_Toc60681738)

[12. Transient EL 20](#_Toc60681739)

[13. Model for the internal quantum efficiency of F8BT:*l*-P6(THS) OLEDs 23](#_Toc60681740)

[14. AFM Images 30](#_Toc60681741)

[References 31](#_Toc60681742)

# Experimental Section

*Spectroscopic ellipsometry*: To obtain the complex refractive index *n+ik*, ellipsometry measurements were carried out using a variable angle spectroscopic ellipsometre (VASE) by J. A. Woollam Co. Inc. with incidence angles ranging from 55° to 65° which were then analysed by means of the commercial software WVASE32®.

*Transient EL characterization*: Time-resolved electroluminescence (EL) measurements were carried out by biasing the device via an Agilent 8114A pulse generator. The pulse width was set at 1 μs, whereas the pulse repetition rate was varied between 49.67 and 208 kHz*.* The photons emitted by the OLEDs were collected by collimating optics and fed into an f/4, 300 mm, Acton Research Spectra Pro SP-2300i triple-turret monochromator. A Hamamatsu H7422-20 Peltier-cooled photosensor module was used as the detector. EL transients were collected at various wavelengths by means of a PicoQuant TimeHarp-100 and a PicoQuant NanoHarp-250 electronic boards, synchronously driven together with the voltage pulser. The time-resolved EL spectra were successively reconstructed by integrating the signal within various time intervals, during the application of the voltage pulse and after its end. In this way, both transients at a fixed wavelength and spectra at different delays could be recorded.

# Transient photoluminescence of the *l*-P*N* oligomers in solution


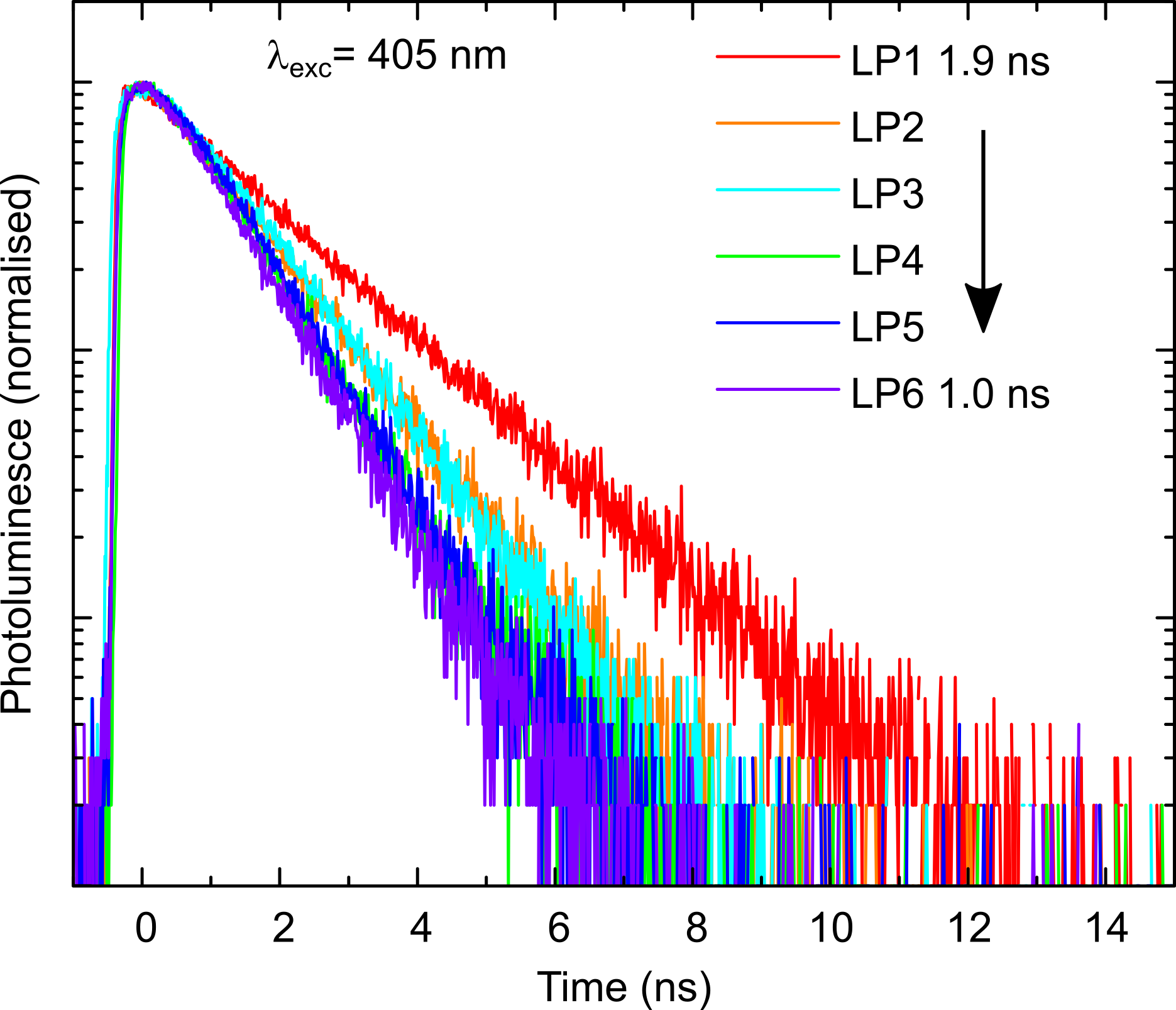


***Figure S1.*** *Photoluminescence (PL) decay spectra of the oligomer series in solution (10^-6^ M in toluene). Normalized PL decay of the l-PN(THS) oligomer series measured via time-correlated single photon counting (TCSPC) at the respective PL maxima. Each PL decay could be fit with a single exponential decay function. In the legend we noted that the extrapolated decay lifetime decreases from $\tau$ = 1.9 ns to $\tau$ = 1.0 ns (± 0.1 ns) with the increase of the oligomer length. Samples were excited with a 405 nm ps pulsed diode laser.*

# Radiative rate calculation


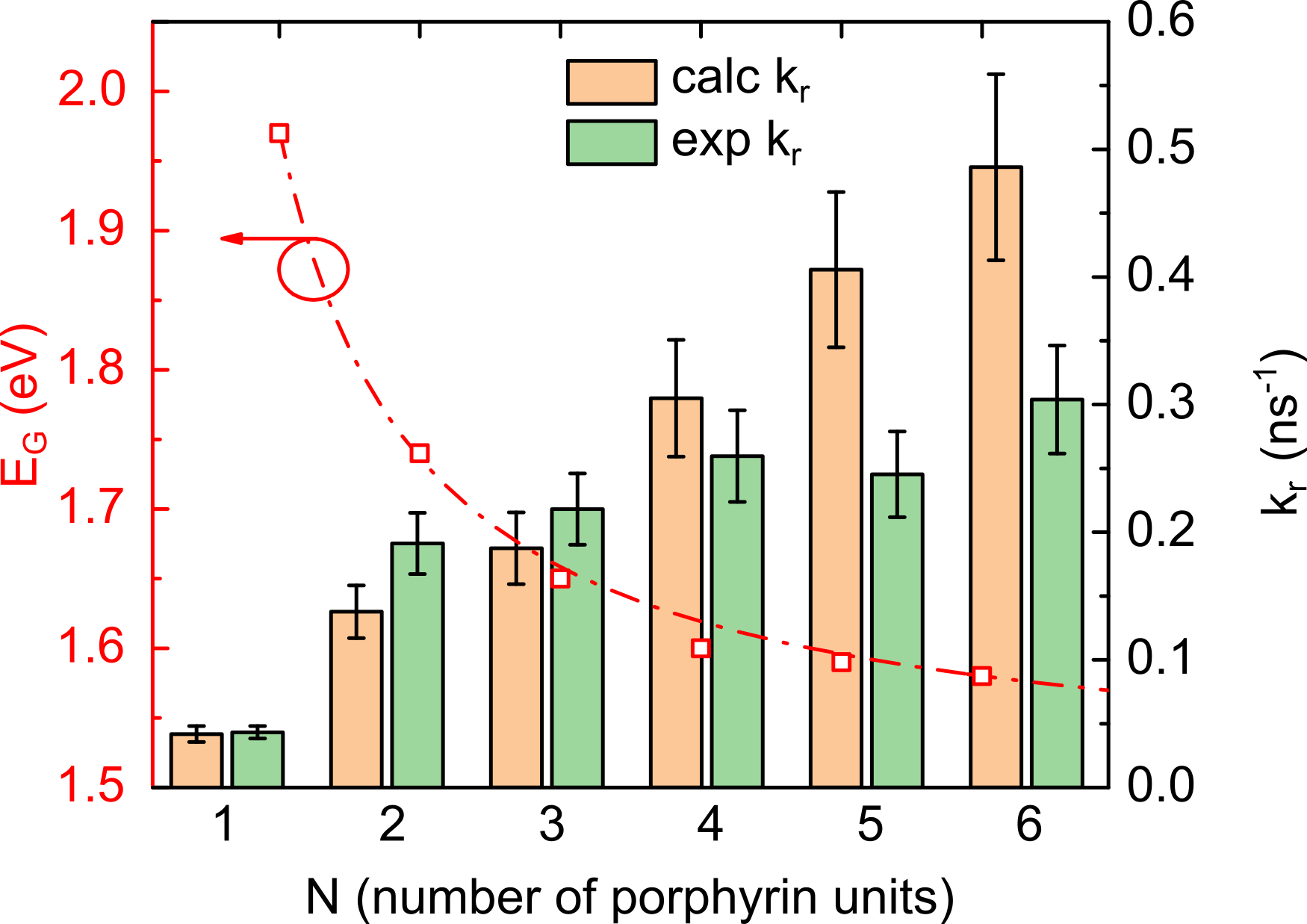


**Figure S2**. Calculated/experimental radiative rates ($k_{r})$ and energy gaps (E_G_) versus number of porphyrin units ($N$). Calculated $k_{r}$ values were obtained via the Strickler-Berg relation reported below^1^ (orange columns) whereas experimental $k_{r}$ (green columns) were extracted from the experimental decay time ($\tau$) and the photoluminescence quantum yield (PLQY) (green columns). Red squares represent the E_G_ of each oligomer, whereas the red curve is the fit of the E_G_ values to the exponential dependence on the oligomer length (E_G_ ∝ N^-1^) expected from theory^2–4^.

The intrinsic (natural) radiative rate reported above vs. the number of porphyrin units *N* was calculated using the Strickler-Berg relation^1^:

$$k_{r}=2.880\times{10}^{-9}n^{2}\left\langle\tilde{\nu}_{f}^{-3} \right\rangle_{Av}^{-1}\frac{g_{l}}{g_{u}}\int\varepsilon dln(\tilde{\nu})$$

where $n$ is the solvent refractive index, $\frac{g_{l}}{g_{u}}$ is the ratio between the degeneracy of the ground and the emitting state (set equal to one, as reported by Duncan et al. for similar Zn‑porphyrin oligomers^5^), $\varepsilon$ is the molar extinction coefficient and $\left\langle\tilde{\nu}_{f}^{-3} \right\rangle^{-1}$ is given by:

$$\left\langle\tilde{\nu}_{f}^{-3} \right\rangle_{Av}^{-1}=\frac{\int I(\tilde{\nu})d\tilde{\nu}}{\int\tilde{\nu}^{-3}I(\tilde{\nu})d\tilde{\nu}}$$

with $I(\tilde{\nu})$ representing the intensity of the fluorescence spectrum, measured in terms of numbers of quanta at each frequency.

The experimental $k_{r}$ (and $k_{nr}$) values were extracted from knowledge of the experimental decay time ($\tau$) and of the photoluminescence quantum yield (PLQY) reported in Figure 2c of the main manuscript, as

$$k_{r}=\frac{PLQY}{\tau}$$

$$k_{nr}=\frac{1}{\tau}-k_{r}$$

As shown in Figure 2d of the main text, $k_{nr}$ extracted from this procedure is nearly constant as a function of the oligomer energy gap, with an absolute value of the slope of ln($k_{nr}$) ~ 0.86 ± 0.16 eV^‑1^, i.e. over one order of magnitude lower compared to those obtained from different series of fluorescent and phosphorescent dyes in previous studies (~ 10 eV^‑1^).^6–8^ This dependence of $k_{nr}$ on *E_G_* is thus unexpected, and we attribute it to either reduced exciton-vibrational coupling or reduced intersystem crossing (ISC), which results from the increasing mismatch of the spatial extent of singlets and triplets with increasing oligomer length *(N).* This is obviously desirable in terms of preventing a drastic decrease of the PLQY with decreasing energy gap as predicted by the *E_G_* law.

Interestingly, we concomitantly observe an increase of $k_{r}$ with *N*, which contributes to the remarkable increase of PLQY up to 31% (for the hexamer). Plotting $k_{r}$ versus *E_G_* as in Figure 2d of the main text, reveals that $k_{r}$ evolves with an approximatively exponential dependence on *E_G_* with a slope of ln($k_{r}$) ~ ‑ 4.67 ± 0.53 eV^‑1^, i.e. six times faster rate compared to ln($k_{nr}$). Notably, such a trend for $k_{r}$ seems to be in contrast with theory, which would predict higher radiative rates for large-gap emitters with respect to those with smaller gaps narrow-gap ones, following approximately a cubic dependence on the emission energy^6^. However, this applies only to series of chromophores in which the oscillator strength of the transition responsible for the emission does not change significantly. As shown in Figure 1a of the main text, the *l*‑P*N* oligomers here investigated exhibit an increase of the oscillator strength of the Q_X_ transition ($\propto$ *N*), which boosts $k_{r}$ and ultimately counteracts the detrimental effect on the PLQY of the (albeit mildly) increasing $k_{nr}$.

We also note in Figure S2 above that the increase of $k_{r}$ is not as expected according to Strickler-Berg (predicting $k_{r}$ ~ approximately proportional to the oscillator strength)^1^. However, by taking into account the error (propagated from the uncertainties of molar extinction and PLQY measurements), the experimental and the calculated $k_{r}$ values differ substantially only for the longest oligomers (*N* > 4). This discrepancy is probably accounted for by the limits of applicability of the Strickler-Berg equation, which are well-established and have been extensively demonstrated in the literature (please see Crosby et al.^9^ for an exhaustive overview). For the *l*‑P*N* oligomers, the main limitation to the validity of such an equation is the tendency of excited twisted conformers to planarise prior to emission^10–12^. Indeed, it has been demonstrated that the natural (calculated) radiative rate and the experimental one can differ for conjugated systems exhibiting intramolecular configurational changes in the excited state^9,13^. Such a limitation applies in particular to long oligomers, which, as discussed in the main text, exhibit a higher torsional disorder in the ground state.

# PLQY of the F8BT:*l*-P6(THS) and TFB: *l*-P6(THS) solid-state blends

***Table S1.*** *PLQY of the F8BT solid-state thin films with different l-P6(THS) loadings.*

| Sample | PLQY  [%] | PL in NIR^a)^  [%] |
| --- | --- | --- |
| F8BT | 54 ± 5 | 3 |
| 0.5 w/w% *l*-P6(THS) | 32 ± 3 | 35 |
| 1.0 w/w% *l*-P6(THS) | 17 ± 2 | 52 |
| 2.5 w/w% *l*-P6(THS) | 13 ± 2 | 62 |
| 5.0 w/w% *l*-P6(THS) | 6 ± 1 | 83 |
| 10.0 w/w% *l*-P6(THS) | 3 ± 1 | 85 |

^a)^ % of photons emitted at *λ* > 700 nm.

***Table S2.*** *PLQY of the TFB solid-state thin films with different l-P6(THS) loadings.*

| Sample | PLQY  [%] | PL in NIR^a)^  [%] |
| --- | --- | --- |
| TFB | 26 | - |
| 0.5 w/w% *l*-P6(THS) | 33 | 47 |
| 1.0 w/w% *l*-P6(THS) | 30 | 60 |
| 2.5 w/w% *l*-P6(THS) | 31 | 64 |
| 5.0 w/w% *l*-P6(THS) | 24 | 71 |
| 10.0 w/w% *l*-P6(THS) | 12 | 90 |

^a)^ % of photons emitted at *λ* > 700 nm.

# Absorption and PL spectra of the TFB:*l*-P6(THS) solid-state blends


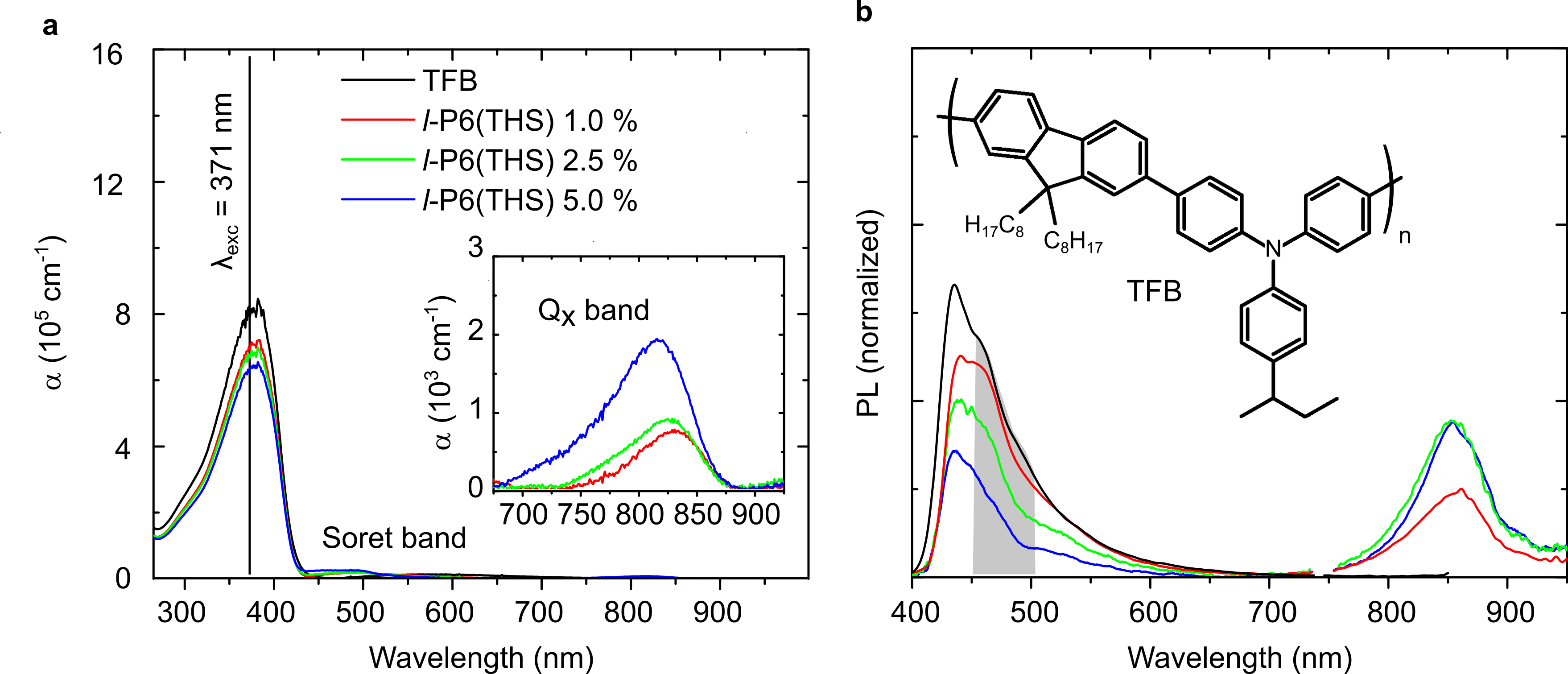


**Figure S3.** Optical properties of TFB:l-P6(THS) blends in solid-state thin films. (a) Absorption spectra of TFB:l-P6(THS) at different hexamer concentration and (b) corresponding normalized PL spectra, obtained by exciting the films with at 371 nm laser diode. The inset in (a) highlights the evolution of the Q_X_ l-P6(THS) absorption component at increasing hexamer concentration. PL spectra in (b) are normalized such as the integrated areas are proportional to the relative PLQY value. The grey shaded area is to highlight the optimal spectral overlap between the TFB emission and the l-P6(THS) Soret band.

# TFB:*l*-P6(THS) OLEDs

As illustrated in Figure S4 and Table S3, TFB:*l*-P6(THS) PLEDs with 2.5 w/w% concentration emit > 80 % of the photons in the NIR region (λ > 700 nm), with EL maximum at 825 nm, thanks to both energy transfer and charge transfer from the polymer to the low gap emitting hexamer sites. The average turn‑on voltage of TFB:*l*‑P6(THS) PLEDs was 4.2 V and the maximum radiance (R_MAX_) observed was 0.2 mW/cm^2^. These devices showed a maximum EQE of 0.13 % at 6.5 V of applied voltage with 10 mA/cm^2^ of current density, with an average maximum EQE of 0.10 %.

Although the EQEs are in agreement with those previously obtained with THS‑free P6 oligomers in F8BT with BP as metal ligand^14^, higher values were expected, given the relatively high PLQY obtained from the TFB solid-state blends (Table S2) compared to the F8BT ones (Table S1). One reason for such low EQEs is probably the 0.5 eV energetic barrier (Figure 1) for electron injection imposed by the high-lying LUMO level of TFB. A second explanation might be related to the anomalous shape of the EL spectra in the visible range, where the TFB blends present not only a residual strong emission from the TFB at 435 nm, but also an additional EL component peaked at 630 nm (Figure S5), not observed in the PL experiments. Interestingly, the energy of this band (1.8 - 2 eV) corresponds to the energy difference between the HOMO of TFB and the LUMO of *l*-P6(THS) (-5.3 ± 0.1 eV and -3.5 ±0.1 eV, respectively, Figure 1 of the main text), suggesting that an intermolecular transition might be responsible for this emission. This interpretation is supported by the “quasi type-II” nature of the TFB:*l*‑P6(THS) heterostructure, given that the mismatch between the TFB and *l*-P6(THS) HOMOs is ~ 0.2 eV, whereas the mismatch between the LUMOs is 1 eV. Such configuration may lead to a higher delocalization of the hole wavefunction inside the blend compared to the electron one, which is better confined in the *l*-P6(THS) low-gap sites. However, it cannot be ruled out that EL band at 630 nm might originate also from oxidative degradation of the TFB, leading to the formation of charge and exciton trapping “keto‑defect sites”^15^.


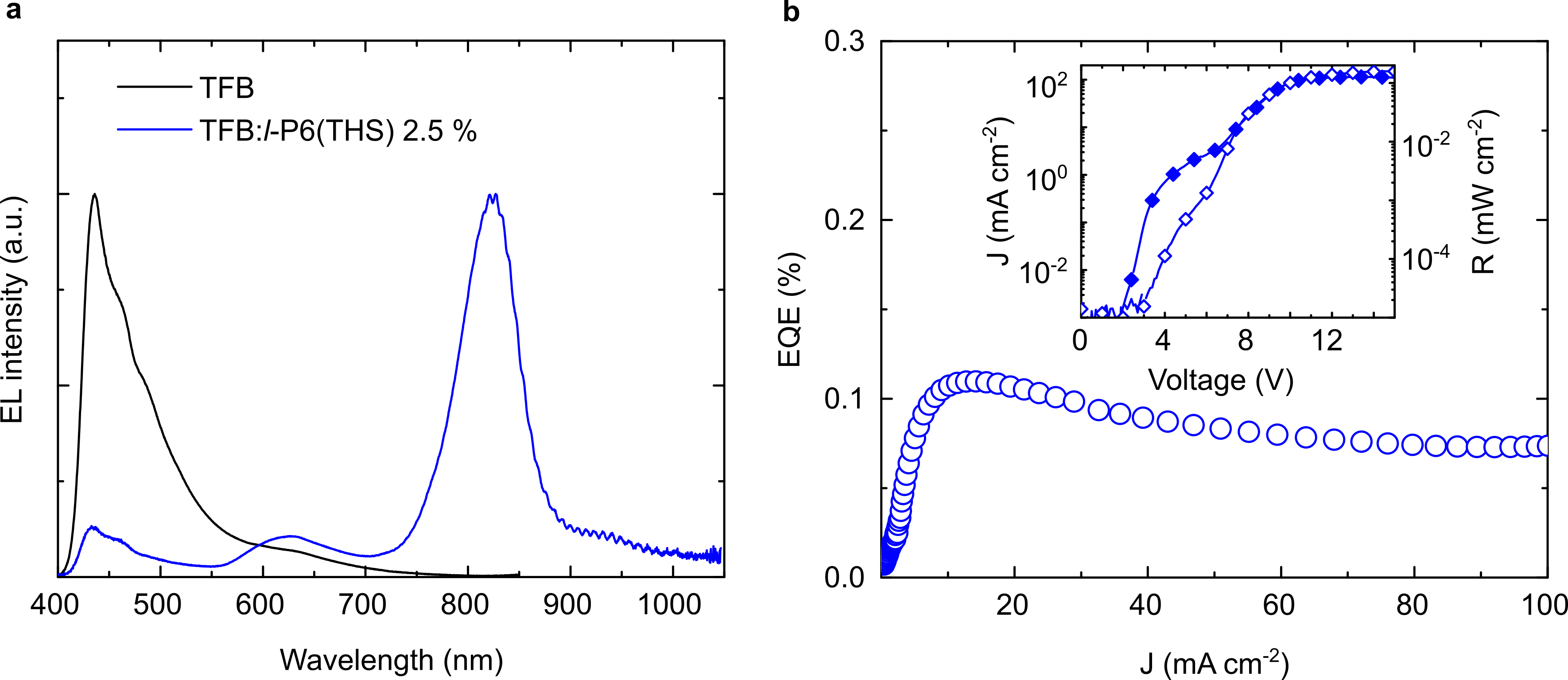


**Figure S4.** (a) EL spectra and (b) EQE versus current density of OLEDs incorporating TFB:l-P6(THS) as active layer. The corresponding JVR curves are shown in the inset.

***Table S3.*** *Summary of the TFB PLEDs electro-optical performance.*

| sample | V_ON_  [V] | <R_MAX_>  [mW/cm^2^] | EQE_MAX_  [%] | <EQE_MAX_>  [%] | EL in NIR  [%] |
| --- | --- | --- | --- | --- | --- |
| TFB:*l*-P6(THS) 2.5% | 4.2 ± 0.4 | 0.2 ± 0.1 | 0.13 | 0.09 ± 0.02 | 87 |

# Absorption and PL of spectra of neat *l*-P6(THS) films


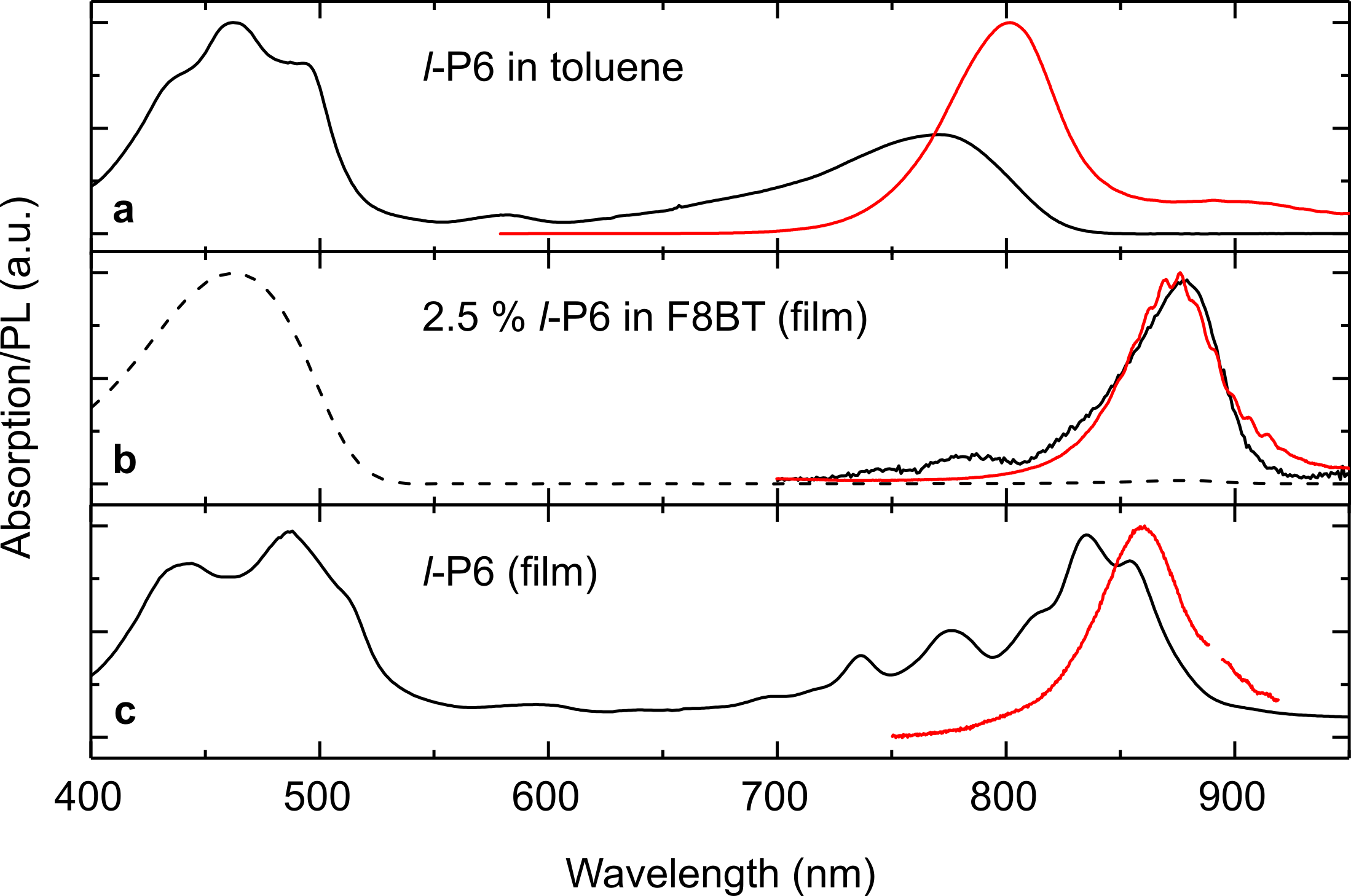


***Figure S5.*** *Normalized absorption (black lines) and PL (red) spectra of (a) l-P6(THS) in toluene solution, (b) in blend with F8BT and (c) in a neat film deposited via spin coating from a toluene solution. In (b), the dashed line represents the absorption spectrum of the F8BT:l-P6(THS) blend, dominated by the F8BT absorption profile peaked at 450 nm. Such a spectrum is zoomed in the 700-950 nm spectral region (full black line) to highlight the l-P6(THS) Q_x_ absorption band.*

# Applied voltage dependence of EL spectra


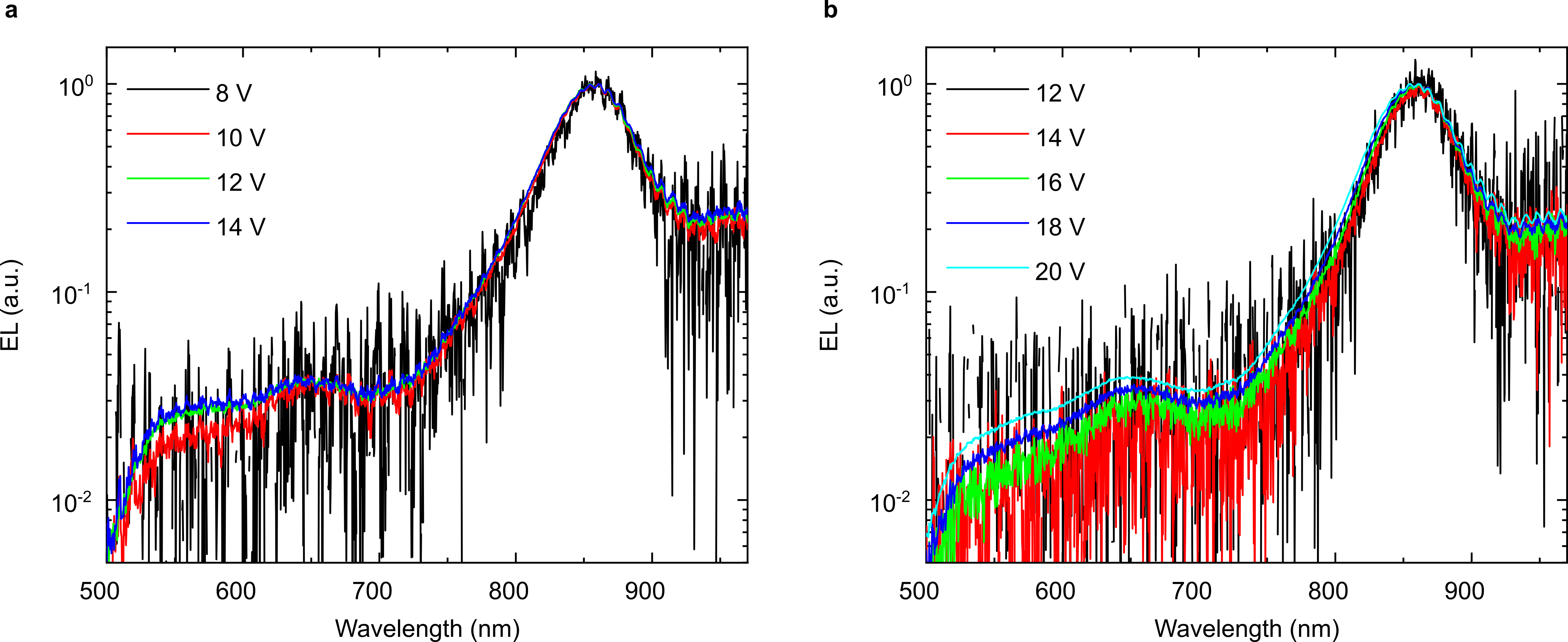


***Figure S6.*** *Normalized EL spectra measured at different applied voltages for F8BT:l‑P6(THS) 2.5 w/w% PLEDs, without (a) and with (b) TFB electron/exciton-blocking layer. Spectra are plotted in semilogarithmic scale to highlight the (weak) bias dependence of the residual emission from the F8BT matrix. Namely, the host emission varies from 3.4 % at 8 V to 5.9 % at 14 V (a) and 1.7 % at 12 V to 5.8 % at 20 V (b). Such a variation may be ascribed to a bias dependence of the branching ratio (b) in Eq. 3 (in section S13 below), i.e. the ratio between the number of excitons directly generated at the oligomer sites and the total number of excitons responsible for the NIR emission, including those resonantly transferred from the F8BT matrix.*

# F8BT:*l*-P6(THS-free) hexamer PLEDs

The relatively high PLQY (~ 30 % in solution, up to 32 % and 33 % in F8BT and TFB blends, respectively) and EL EQE (~ 1 % on average and maximum values up to 3.8 % for PLEDs based on F8BT) of *l*-P6(THS) hexamer, reported in Table S1, S2 and Table 1 of the main text, are possible thanks to the large steric hindrance offered by the THS sidechains, which limits aggregation quenching in favour of radiative recombination. The values we obtained from *l*-PN(THS) toluene solutions are in good agreement with values reported for *l*-PN oligomers with smaller side groups in place of THS.^16^ However, oligomers with shorter sidechains suffer from aggregation quenching also at low concentration. For this reason, previously reported *l*‑PN PLQY values were achieved by dissolving the oligomers in toluene/pyridine mixtures,^16,17^ since it is well known that pyridine coordinates the Zn centres and minimizes the effects of aggregation.

Compared to the previously reported “THS-free” linear P6 with no metal ligands in the active layer, the average EQE achieved with *l*-P6(THS) is one order of magnitude higher.^14^ In the previous work,^14^ the meso-aryl groups where functionalized with 3,5-bis(octyloxy)phenyl (OCT) sidechains, which are too small to prevent aggregation quenching. For this reason, the best trade-off between emission efficiency and efficient energy/charge transfer to the hexamer was found by adding 3 w/w% of 4‑benzylpyridine (BP) metal coordinating agent to a F8BT:*l*‑P6(OCT) blend containing 10 w/w% of hexamer, yielding a maximum EQE of only 0.1 %

To reliably compare the performances of *l*-P6(OCT) and *l*-P6(THS) devices, we tested again the EL performances of a new batch of *l*-P6(OCT), following the same fabrication protocols and using the same F8BT matrix used in the present work for *l*‑P6(THS). In analogy with the *l*-P6(THS), we tested *l*-P6(OCT) blends with up to 10 w/w% and achieved the best performances from F8BT:*l*-P6(OCT) blends with 0.5 w/w% of hexamer. In Table S4 we quantitatively compare the optoelectronic properties of the best performing F8BT:*l*-P6(THS) and :*l*-P6(OCT) blends. In Figure S7 the EL spectra, JVR curves and EQE versus J curves are reported.

A clear hint of oligomer aggregation in F8BT:*l*-P6(OCT) 0.5 w/w% blend is its red-shifted EL spectrum, which is peaked at 900 nm and ~ 50 nm shifted in the NIR with respect to the one of F8BT:*l*-P6(THS) 2.5 w/w% blend. Furthermore, we had to limit the concentration of the *l*-P6(OCT) oligomer in the F8BT matrix down to 0.5 w/w% to obtain efficiencies > 0.3 %, measuring a maximum EQE of 1.2 %. Such an efficiency is two times lower compared to the maximum EQE measured from F8BT:*l*-P6(THS) 2.5 w/w% devices (Table 1 and Table S4). In addition to this, as reported in Table S4, the average maximum radiance is six times lower compared to the one obtained with *l*-P6(THS).

***Table S4.*** *Summary of the OLEDs electro-optical performance with and w/o THS.*

| sample | V_ON_  [V] | <R_MAX_>  [mW/cm^2^] | EQE_MAX_  [%] | EL in NIR^a)^  [%] |
| --- | --- | --- | --- | --- |
| 2.5% THS | 6.6 ± 1.2 | 0.30 ± 0.10 | 2.25 | 98 |
| 0.5% OCT | 6.8 ± 0.3 | 0.05 ± 0.02 | 1.17 | 95 |

^a)^ % of photons emitted at *λ* > 700 nm.


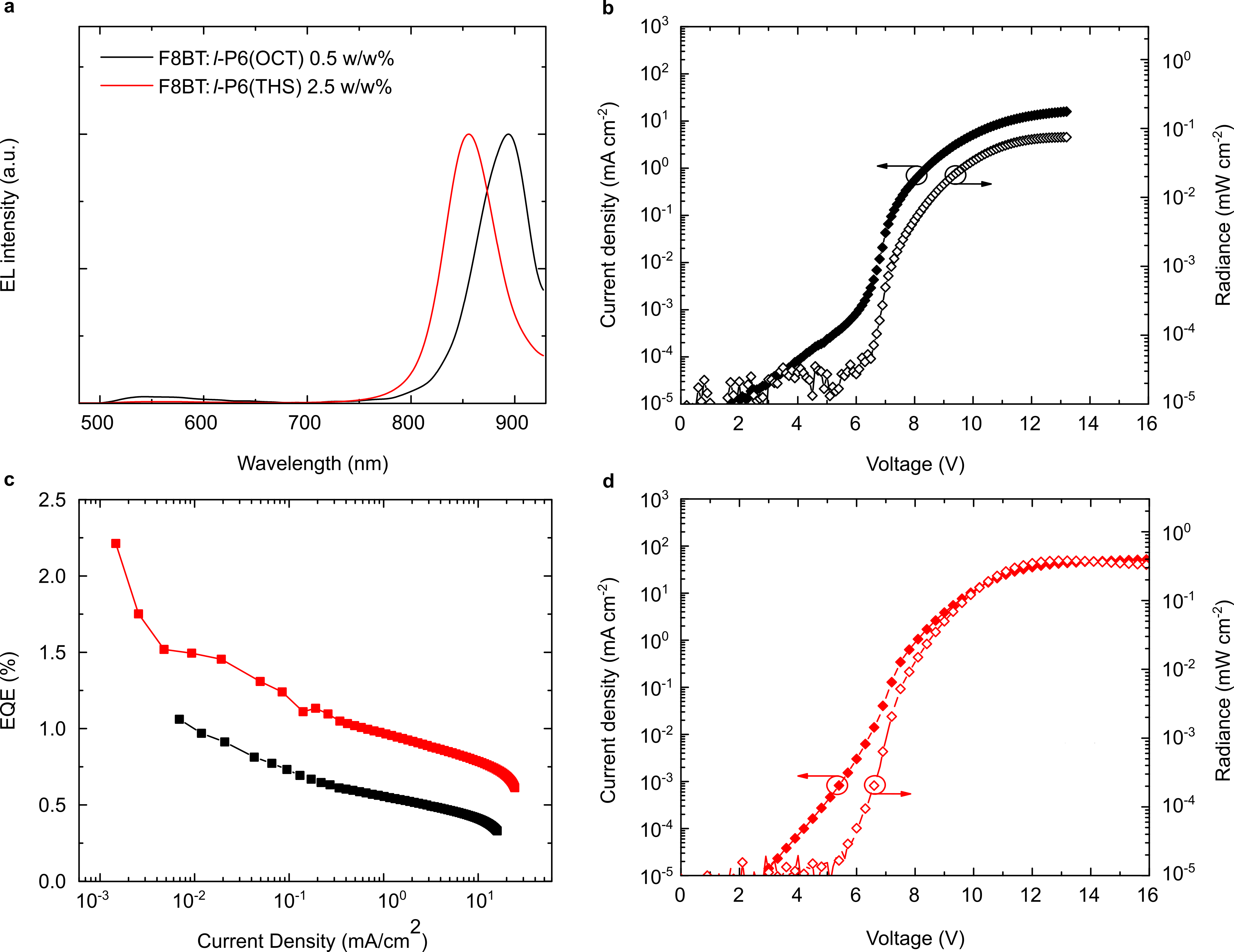


***Figure S7****. The effect of the sidechains on the PLEDs performance. Normalised EL spectra (a), EQE vs J (c) and JVR curves (b,d) of the best performing F8BT:l-P6(OCT) 0.5 w/w% (black lines) and F8BT:l-P6(THS) 2.5 w/w% (red lines) PLEDs.*

# Refractive indices via spectroscopic ellipsometry


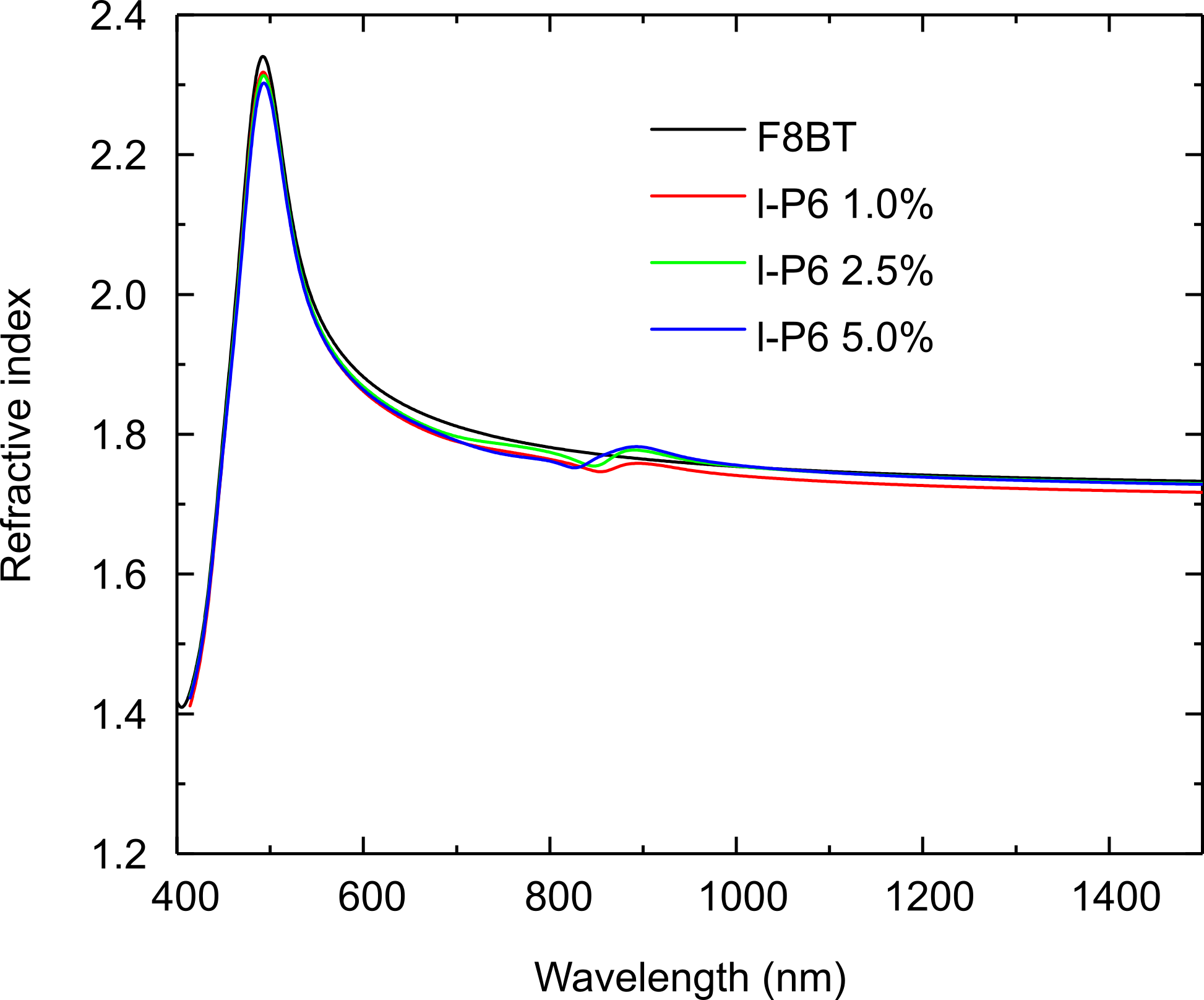


***Figure S8.*** *Refractive index dispersions for neat F8BT, F8BT:l-P6(THS) blends at different hexamer concentration and neat l-P6(THS) in solid-state thin films in the 400-1500 nm spectral range. Ellipsometric spectra of neat F8BT and neat l-P6(THS) were fit with a combination of four and eight Lorentzian oscillators, respectively, reproducing analytically also the F8BT and l-P6(THS) absorptance spectra. Dispersions of the complex refractive indices of F8BT and l-P6(THS) were used to model an effective material accounting for the ratio in the blend of F8BT and l-P6(THS) and fit the blends ellipsometric spectra.*

# Neat F8BT PLEDs and efficiency roll-off


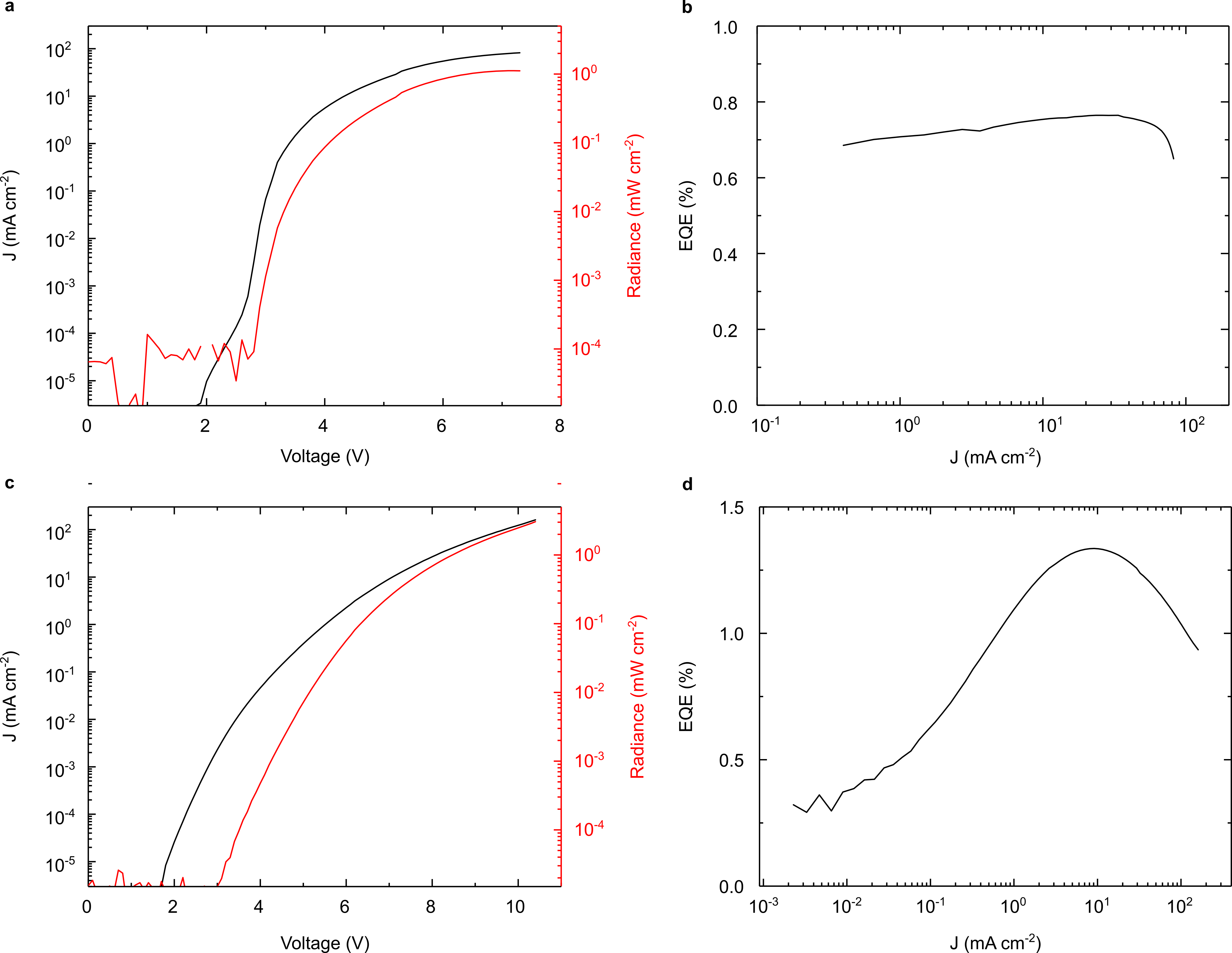


***Figure S9.*** *JVR (a,c) and EQE versus current density (c,d) curves of neat F8BT devices, without (a,b) and with TFB electron/exciton-blocking layer (c,d).*

As shown in Figure S9, we found that both types of our NIR OLEDs (without and with TFB EBL, see Table 1 and Figure 4 for comparison) outperform our control F8BT ones, which exhibit maximum EQEs of 0.75 % (1.32 % for samples with TFB EBL) at ~ 10 mA/cm^2^. Although these devices were not optimised (e.g. in terms of thickness)^18^, they were prepared exactly in the same conditions as the blend ones, and with the same F8BT used as the host in there, and thus serve to rule out that the high efficiency of NIR devices at low voltage (whose spectra are notoriously hard to measure, given the low signal to noise ratio, S/N, in such operating conditions - see Figure S6) might be due to F8BT emission.

Looking at the EQE versus current plots, the EQE of the control devices (Figure S9b,d) increases until a current density J ~ 10 mA/cm^2^, beyond which value the radiance eventually saturates and the efficiency drops, as also shown previously, as a result of a complex interplay between triplet to singlet conversion (TTA and TADF) and singlet-triplet annihilation (STA), among other processes^18,19^.

For the NIR OLEDs (Figure 4 of the main manuscript), we suggest that the roll-off (starting at lower J, but similar voltages compared to neat F8BT ones) results from a combination of different exciton quenching processes, such as progressive “saturation” of the (porphyrin) exciton recombination centres, STA and alteration of the charge-carrier balance with current density^20^. Expectedly, as shown in Fig. S10 (see next section), we also observed delayed EL from our neat and F8BT:*l*-P6(THS) OLEDs, confirming the presence of triplet to singlet conversion pathways.

# Transient EL

To explore and prove the presence of TTA in our blended devices, we also carried out time-resolved EL measurements by driving the OLEDs in a voltage‑pulse mode. In Figure S10a we report in particular the EL spectra averaged over the temporal windows detailed in figures S10b. We observed no variation of the spectra over time, and remarkably we also detected EL in the 4^th^ interval (8‑18 μs) after the voltage pulse end. Such a delayed EL does not originate from the residual current after the switch-off of the driving pulses, as no current could be measured already at ~ 1 μs after the pulse end (Figure S10c). We attribute such delayed fluorescence to triplet-to-singlet conversion via either triplet-triplet annihilation (TTA) or thermally‑activated reverse intersystem crossing (RISC)^18,19^. Remarkably, there is little or no variation of the spectral shape in the different intervals, but there is a clear (albeit slightly noisy) contribution to the spectra in the F8BT region, thereby confirming that that delayed emission is caused by TTA/RISC occurring primarily on F8BT, with subsequent NIR emission deriving from Förster resonance energy transfer (FRET) to the porphyrin oligomers.

**
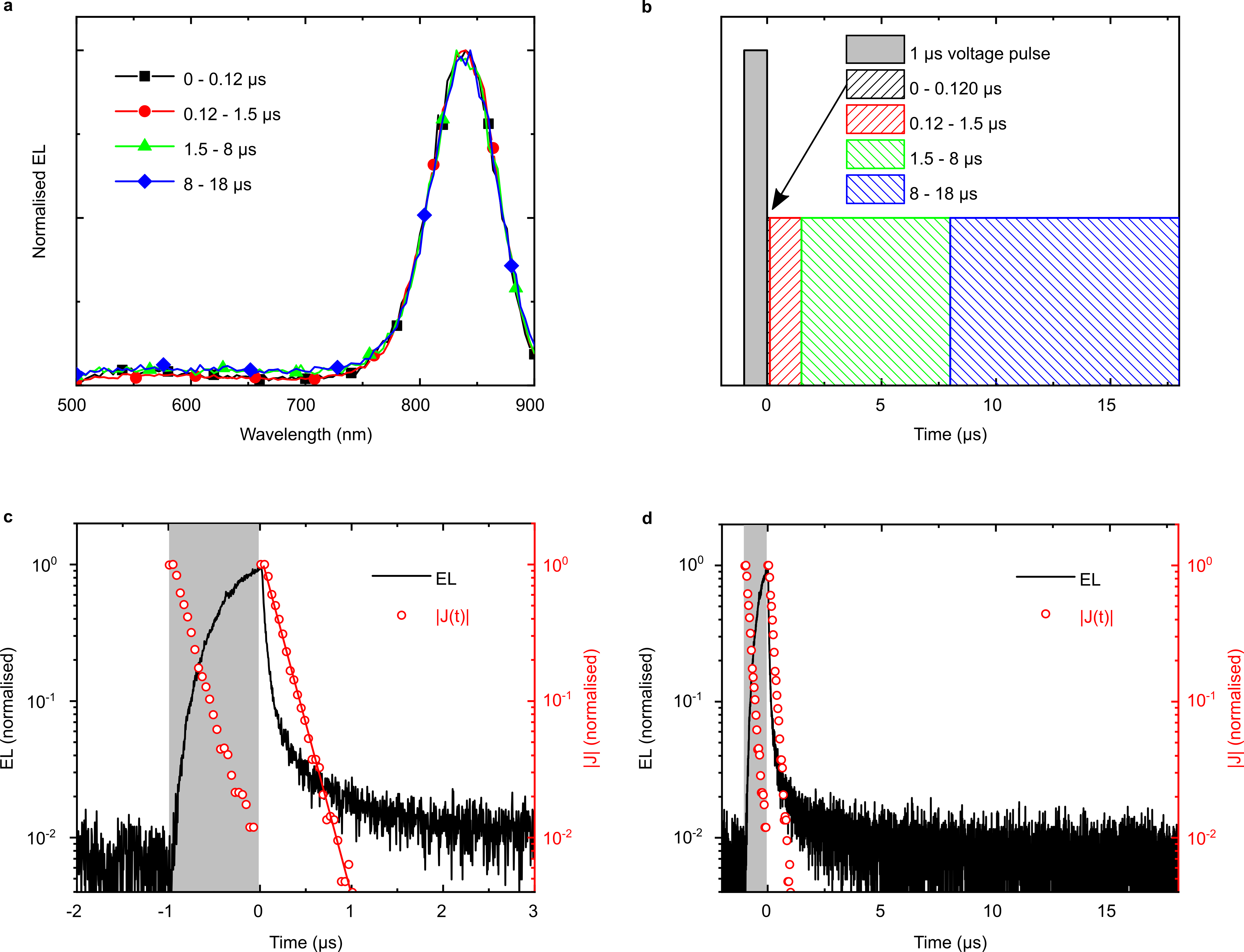
**

***Figure S10.*** *(a) Normalized spectra collected from an F8BT:l-P6(THS) 2.5 w/w% OLED by integrating the EL signal within four time intervals after the end of a 10 V rectangular voltage pulse (1 μs pulse width, 49.63‑208 kHz repetition rate). (b) Scheme illustrating the time intervals after the end of the voltage pulse (also reported as a solid grey histogram) during which the time-gated EL spectra have been measured.* *(c,d) Semi-log plot of the transient EL (at 830 nm, black line) up to 3 μs (c) and 18 μs (d) after the pulse end. The red circles represent the absolute value of the transient current (|J(t)|, normalized to its maximum) flowing across the circuit during the OLED pulsed operation. This signal was obtained by measuring the transient voltage drop across a calibrated resistor (R = 47 Ω, with a GWINSTEK GDS-2204 oscilloscope) in series with the device. Such a signal, which is proportional to |J(t)|, decays exponentially (linearly in the semi-log plot), as typically observed when a capacitor is discharged through a resistor.*


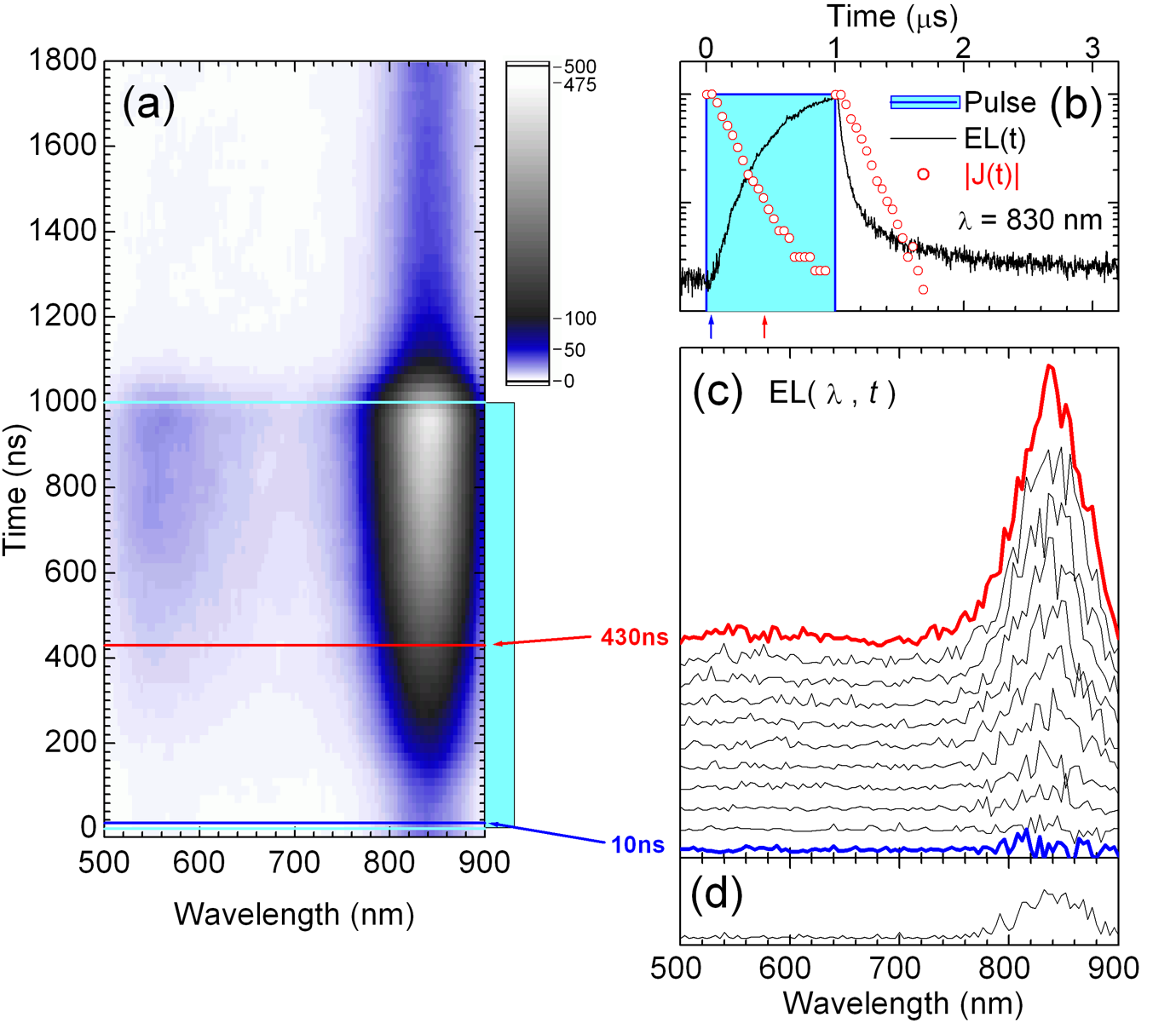


***Figure S11.*** *(a) Intensity map: time evolution of the EL spectrum of an F8BT:l-P6(THS) 2.5 w/w% OLED biased by 10 V, 1000 ns wide voltage pulses, at a repetition rate of 208 kHz. The voltage pulse is applied between t = 0 ns and t = 1000 ns (cyan box). The EL intensity (a.u.) is represented via a false color scale as shown on the top right. A 5-point adjacent averaging has been applied to the data. (b) Semi-log plot of the transient EL(t) (black line), as detected at the (fixed) wavelength of λ = 830 nm during, and up to 2.2 μs after the end, of a 1 μs-wide, 10 V rectangular pulse (cyan box). Red circles: absolute value of the transient current (|J(t)|, normalized to its maximum) flowing across the circuit during the OLED pulsed operation. (c) The time-evolution of electroluminescence from the OLEDs in the early stages following the 10 V pulse onset. EL spectra, collected in eleven successive 20 ns-wide time intervals centered from 10 ns to 430 ns after the pulse rising edge, are shown. Two successive spectra are time-separated by 40 ns. The long-lived emission component (d) still measurable on a 20 ns-wide time interval just before the pulse rise has been subtracted from all the other data. The spectra are vertically shifted for the sake of clarity. The blue and the red arrows point to the corresponding position, on the time scale, both on graphs (a) and (b).*

# Model for the internal quantum efficiency of F8BT:*l*-P6(THS) OLEDs

The internal quantum efficiency (IQE) of an OLED has usually been expressed as:

$IQE=\gamma r_{st}\eta$ (1)

where $\gamma$ is the electron-hole balance factor, $r_{st}$ is the singlet to total number of excitons ratio, and $\eta$ the PL efficiency. The IQE can be evaluated from the experimentally measured EQE, from the relation:

$EQE=\xi IQE$ (2)

with $\xi$ representing the photon out-coupling efficiency.

In the past, equation (1) and (2) above have often been used to estimate a lower bound for $r_{st}$ and compare it to the maximum expected according to simple spin statistics arguments (0.25) under the hypothesis of spin-unpolarized charge injection from the electrodes, also with a view to verify the assumption that the exciton formation probability is the same for both singlets and each of the (three) triplet states^21^. Under the conservative assumption $\gamma=1$, and with additional knowledge of EQE, $\eta$, and $\xi$ (e.g. to be conservatively estimated by considering emitting chromophores to be oriented parallel to the device plane so that $\xi=0.75/n^{2}$, and by determining $n$ via spectroscopic ellipsometry - see Figure S8) the data we report in table 1 would yield $r_{st}=$ 50 ± 5 % and 30±3 % for our diodes with and without the TFB layer respectively, and would thus point to a different probability of formation of singlets and triplets.

However, this model would disregard the possibility that conversion of triplets to singlets via triplet-triplet annihilation (TTA) and/or reverse intersystem crossing (RISC - also indicated as thermally activated delayed fluorescence or TADF), can actually increase $r_{st}$. Such a possibility has in fact been reported and discussed in a number of studies^18,19,22^, that among other aspects have shown that the singlets regeneration from TTA can only contribute an additive factor of 0.15 on top of the 0.25 expected from the simple spin statistics^19^. A recent analysis of diodes incorporating very thick (~ 1.2 μm or so) neat F8BT layers has reported a singlet generation yield of ~ 30%, explained by the presence of TADF.

In OLEDs having a host-guest blend as the active layer, such as the F8BT:*l*‑P6(THS) devices investigated in this work, the IQE can be considered as the sum of three contributions: one from singlets that decay in the F8BT host (${{IQE}_{F}}^{*}$), a second one deriving from (electrically-generated) singlet excitons formed initially on F8BT and then transferred onto the *l*-P6(THS) via FRET (${{IQE}_{P6}}^{ET}$), and a third contribution from singlets directly generated by mutual hole-electron capture at the *l*‑P6(THS) sites (${{IQE}_{P6}}^{*}$). A fourth fraction corresponding to the singlets potentially generated by conversion of triplets directly formed on the guest (via mutual hole-electron capture) into singlets is ignored here in consideration of the low loading (few % at maximum) of the blends considered here. We also exclude reverse transfer of excitons (either singlets or triplets) from the guest to the host, because of the sizeable difference in respective singlet and triplet energy levels between host and guest.

By rescaling the IQE for the fraction of photons emitted by *l‑*P6(THS) ($\rho_{NIR}$), we can discard ${{IQE}_{F}}^{*}$ and obtain the “NIR IQE” (*IQE_NIR_*):

${IQE}_{NIR}=\rho_{NIR} IQE=$

${IQE}_{NIR}={{IQE}_{P6}}^{ET}+{{IQE}_{P6}}^{*}$

Similar relations would hold among the respective EQEs under the hypothesis that the outcoupling factors are the same for all terms.

To get further insight we now express the terms above as a function of relevant parameters and observables. To this end we further assume that the injected charges branch between F8BT and *l*-P6(THS), with a ratio *b* in favour of the hexamer sites. In addition, we will also indicate with $\gamma_{F}$ ($\gamma_{P})$ the electron-hole balance factor for F8BT (*l*-P6(THS)), and with $\eta_{F}$ ($\eta_{P6}$) the PL efficiency of F8BT (*l*-P6(THS)). We assume no phosphorescence (i.e. *η*_phos_ = 0) however, we allow for triplets to (re)generate singlets via TTA or thermally-activated reverse intersystem crossing. Therefore, we indicate with $r_{stF}^{*}$ and $r_{stP6}^{*}$ the "effective" singlet to total number of excitons ratio, *i.e.* including the contribution from singlets generated in F8BT via TTA or thermally-activated RISC. Finally, $\eta_{ET}$ stands for the efficiency of energy transfer. We can thus write:

${IQE}_{NIR}=\left[ \left( 1-b \right)\gamma_{F}r_{stF}^{*}\eta_{ET}\eta_{P6}+b{\gamma_{P6}r}_{stP6}^{*}\eta_{P6} \right]$ (3)

And from Equation 3, we can derive:

${\gamma_{P6}r}_{stP6}^{*}=\frac{1}{b\eta_{P6}}\left[ {IQE}_{NIR}-(1-b){\gamma_{F}r}_{stF}^{*}\eta_{ET}\eta_{P6} \right]$ (4)

or, conservatively assuming the electron-hole balance at the hexamer sites to be ideal (i.e. $\gamma_{P6}$ = 1):

$r_{stP6}^{*}=\frac{1}{b\eta_{P6}}\left[ {IQE}_{NIR}-(1-b){\gamma_{F}r}_{stF}^{*}\eta_{ET}\eta_{P6} \right]$ (5)

which gives us $r_{stP6}^{*}$ as a function of factors that can be measured or estimated from measurement (such as the IQE).

We summarize all the experimentally derived parameters and $r_{stP6}^{*}$ for the best performing F8BT:*l*‑P6(THS) 2.5 % OLEDs (without and with the TFB interlayer) in Table S5.

Here, ${IQE}_{NIR}$ is obtained by using the conventional relation for EQE (Equation 2), with $\xi$ = 0.24, considering a refractive index of 1.77 at 850 nm (as measured from ellipsometry, Figure S9) and conservatively assuming in-plane dipoles^18,23^.

The product $\gamma_{F}r_{stF}^{*}$ reported in Table S5 has been extracted from the characterization of the neat F8BT devices (Figure S6), by using Equation 2 with $\xi$ = 0.20 (for in-plane dipoles^18,23^ and a refractive index of 1.95 at 560 nm, Figure S9) and $\eta_{F}$ = 0.54, as reported in Figure 2 of the manuscript.

The energy transfer efficiency $\eta_{ET}$ has been obtained from the quenching of the F8BT PL when mixed with *l*-P6(THS). From the TCSPC measurements carried out on the F8BT:*l*-P6(THS) 2.5 w/w% blend and undoped F8BT film (Figure S10), we obtain a transfer efficiency $\eta_{ET}=1-\tau_{F*}{/\tau}_{F}$ = 0.82, with $\tau_{F*}$ and $\tau_{F}$ corresponding to the lifetime of the F8BT emission for the blend and the neat films, respectively.

***Table S5.*** *Parameters experimentally derived or extracted from Equation 1-8: maximum EQE (EQE) of F8BT:l‑P6(THS) 2.5 % OLEDs; fraction of photons emitted by l-P6(THS) ($\rho_{NIR}$); EQE rescaled for ρ_NIR_ (to yield EQE_NIR_) and for (1 - $\rho_{NIR}$) (to yield EQE_F_*); EQE of neat F8BT devices (EQE_F_); outcoupling efficiency ($\xi$); PL efficiency of l‑P6(THS) ($\eta_{P6}$) and F8BT($\eta_{F}$); energy transfer efficiency ($\eta_{ET}$); charge branching ratio (b); "effective" singlet to total number of excitons generated on lP6(THS) sites (r_stP6_*).*

| parameter | w/o EBL [%] | with EBL [%] |
| --- | --- | --- |
| EQE | 2.25 | 3.80 |
| $\rho_{NIR}$ | 96.6 | 98.3 |
| EQE_NIR_ | 2.17 | 3.74 |
| EQE_F*_ | 0.08 | 0.06 |
| EQE_F_*^neat^* | 0.75 | 1.32 |
| $\xi_{850 nm}$ | 19.7 | |
| $\xi_{560 nm}$ | 23.9 | |
| IQE_F*_ | 0.39 | 0.33 |
| IQE_F_*^neat^* | 3.80 | 6.69 |
| $\eta_{P6}$ | 31 | |
| $\eta_{F}$ | 54 | |
| $\eta_{ET}$ | 81.5 | |
| b | 44.8 | 73.5 |
| r_stP6_* | 63.2 | 67.3 |


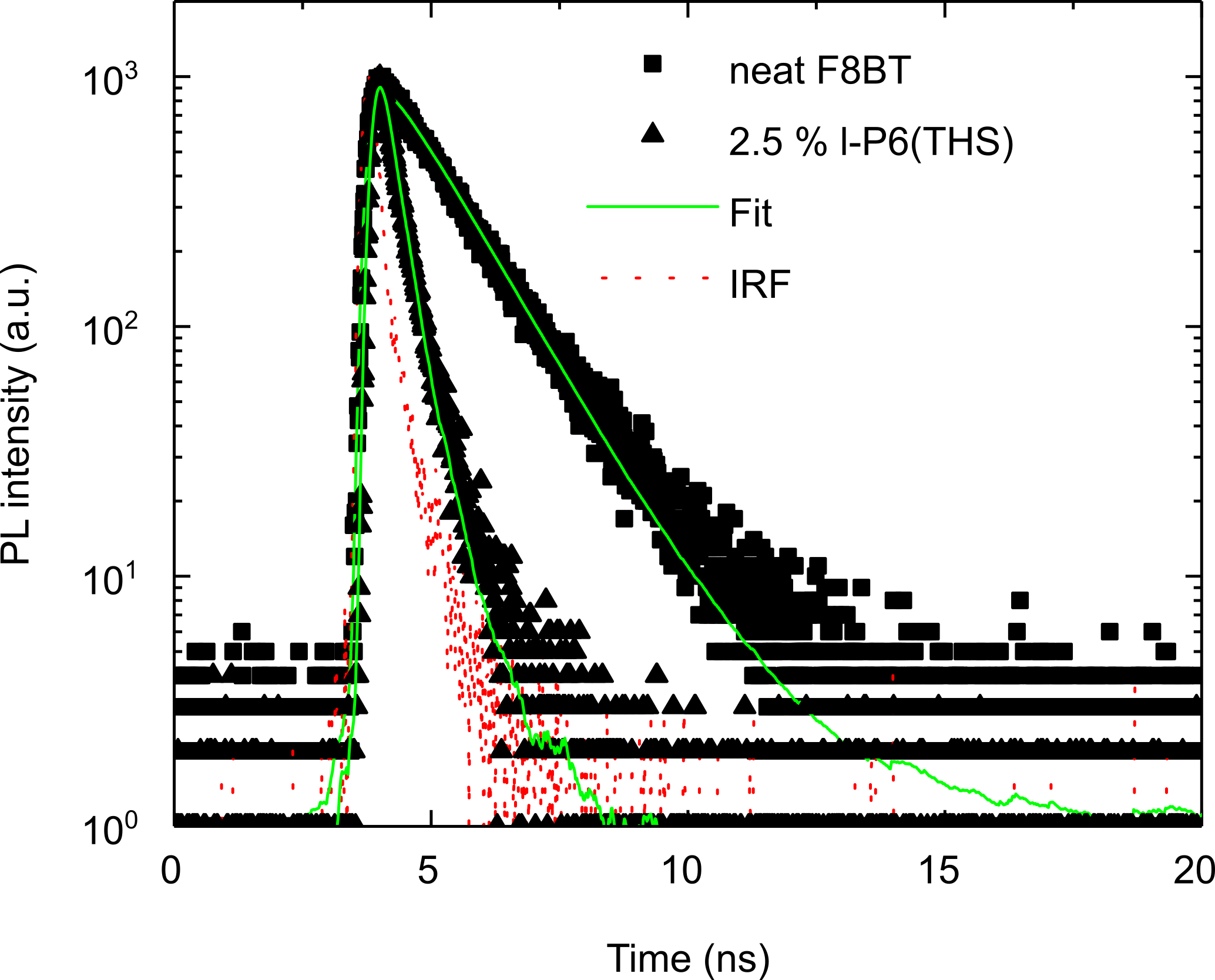


***Figure S12.*** *PL decays of a neat F8BT film and F8BT:l-P6(THS) 2.5 % blend at 560 nm (F8BT PL maximum), measured in air at room-temperature. Each decay could be fit with a single exponential decay function (green line), with $\tau_{F}$ = 1.19 ns (neat film) and  = 0.22 ns (blend) corresponding lifetime. Samples were excited with a 445 nm ps pulsed laser. The red dotted line represents the instrument response function.*

For the same blend, we determined also the branching ratio *b* from the expression of ${{IQE}_{F}}^{*}$ (e.g. the fraction of the measured total IQE due to the residual emission from F8BT) as a function of the parameters defined above:

${{IQE}_{F}}^{*}=\left( 1-b \right)\gamma_{F}r_{stF}^{*}\left( 1-\eta_{ET} \right) \eta_{F}$ (6)

It is worth noting that the product $\gamma_{F}r_{stF}^{*}\eta_{F}$ corresponds to the IQE of the OLEDs with a neat F8BT active layer, if we assume that $\gamma_{F}$ is the same in both blended and neat layers. The EQE of the neat F8BT devices was experimentally measured and denoted as ${{EQE}_{F}}^{neat}$ in Table S5. We assume that the outcoupling factor is the same for both the neat and the blended devices the ratio of the quantum efficiency for the blend and neat devices. Within this straightforward approximation we can then write:

${{IQE}_{F}}^{*}=(1-b)(1-\eta_{ET}){{IQE}_{F}}^{neat}$ (7)

As expected, ${{IQE}_{F}}^{*}\to0$ if $\eta_{ET}\to1$. However, we can rule out complete ET as we do see F8BT emission in the PL and EL spectra, so we can divide by $\left( 1-\eta_{ET} \right)$ and obtain for *b* the following expression:

$b=\left( 1-\frac{{{IQE}_{F}}^{*}}{(1-\eta_{ET}){{IQE}_{F}}^{neat}} \right)=\left( 1-\frac{{{EQE}_{F}}^{*}}{(1-\eta_{ET}){{EQE}_{F}}^{neat}} \right)$ (8)

Finally, the last parameter necessary to obtain $r_{stP6}^{*}$ from Equation 5 is $\eta_{P6}$, which can be approximated to the PL efficiency of the hexamer when blended in F8BT. In principle $\eta_{P6}$ could be obtained by selectively exciting the (planar) *l*‑P6(THS) moieties and by measuring the quantum yield. However, such a procedure is limited by the low concentration of *l*‑P6(THS) and the sensitivity of the spectrometer, and would lead to inaccurate determination for a low-concentration blend as in our case. For this reason, as shown in Table S5, we (conservatively) approximated $\eta_{P6}$ to the 0.31 PLQY measured from the *l*-P6(THS) toluene solution (in practice we expect it to be slightly lower because of residual aggregation and quenching that even the presence of THS chains and blending cannot suppress). The conservative nature of this approximation means that the estimate of $r_{stP6}^{*}$ is in fact a lower bound.

A fully conservative (but unrealistic) estimate which in addition to an overestimated $\eta_{P6}$ = 0.31, and perfect electron-hole balance ($\gamma$ =1), would also assume ideal distance (and orientation) from the reflecting electrode, so as to ensure optimal out-coupling efficiency (*ξ* ~ 1.2/n^2^ ~0.38), would set an even lower limit for $r_{stP6}^{*}$ of 0.4 for the best performing device without EBL, and 0.42 for the one with EBL.

While it is not possibly to completely rule out, on the basis of the current data, the possibility that such ideal conditions are all present in our devices, the simultaneous occurrence of these is rather unlikely, and we propose therefore that it is preferable to take $\xi=0.75/n^{2}$ to capture the device physics and the implication of the data. Taken together, our results thus point to a singlet population well over 40% of the total excitons (the value that had been derived by Wallikewitz et al.,^19^ as the sum of the 25 % initially generated singlets and 15 % additional singlets generated via a chain of consecutive TTA reactions only) thus implying a significantly greater contribution of RISC in addition to or in place of TTA.

Last but not least, we note that while there is no need to invoke “direct” singlet generation over the spin statistical limit (25%) of the total excitons, our data cannot exclude it either, and could in fact be accounted for by this, possibly also in combination with TTA and RISC.

# AFM Images


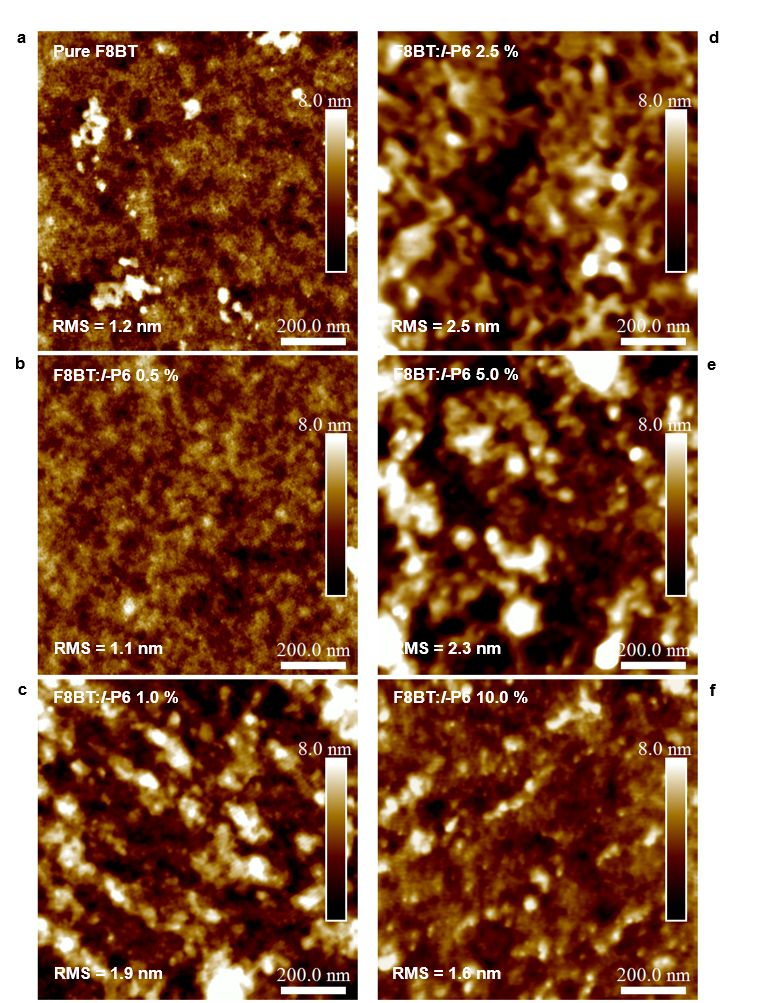


**Figure S13.** *Peak force tapping AFM images of neat F8BT polymer films and relative blends with l-P6(THS). The AFM images have been acquired with a Bruker Dimension Icon AFM. The root-mean-square (RMS) roughness of each film is reported in the corresponding image.*

# References

1. Strickler, S. J. & Berg, R. A. Relationship between Absorption Intensity and Fluorescence Lifetime of Molecules. *J. Chem. Phys.* **37**, 814 (1962).

2. Kuhn, H. The Electron Gas Theory of the Color of Natural and Artificial Dyes: Problems and Principles. in *Fortschritte der Chemie Organischer Naturstoffe / Progress in the Chemistry of Organic Natural Products* 169–205 (Springer, Vienna, 1958). doi:10.1007/978-3-7091-8047-1_5

3. Kuhn, H. The Electron Gas Theory of the Color of Natural and Artificial Dyes: Applications and Extensions. in *Fortschritte der Chemie Organischer Naturstoffe / Progress in the Chemistry of Organic Natural Products* (ed. Zechmeister, L.) 404–451 (Springer, Vienna, 1959). doi:10.1007/978-3-7091-8052-5_9

4. Wenz, G., Müller, M. A., Schmidt, M. & Wegner, G. Structure of Poly(Diacetylenes) in Solution. *Macromolecules* **17**, 837–850 (1984).

5. Duncan, T. V, Susumu, K., Sinks, L. E. & Therien, M. J. Exceptional near-infrared fluorescence quantum yields and excited-state absorptivity of highly conjugated porphyrin arrays. *J. Am. Chem. Soc.* **128**, 9000–9001 (2006).

6. Caspar, J. V, Kober, E. M., Sullivan, B. P. & Meyer, T. J. Application of the energy gap law to the decay of charge-transfer excited states. *J. Am. Chem. Soc.* **104**, 630–632 (1982).

7. Caspar, J. V. & Meyer, T. J. Application of the energy gap law to nonradiative, excited-state decay. *J. Phys. Chem.* **87**, 952–957 (1983).

8. Wilson, J. S. *et al.* The energy gap law for triplet states in pt-containing conjugated polymers and monomers. *J. Am. Chem. Soc.* **123**, 9412–9417 (2001).

9. Crosby, G. A., Demas, J. N. & Callis, J. B. Absolute quantum efficiencies. *J. Res. Natl. Bur. Stand. Sect. A Phys. Chem.* **76A**, 561 (2012).

10. Chang, M. H., Hoffmann, M., Anderson, H. L. & Herz, L. M. Dynamics of excited-state conformational relaxation and electronic delocalization in conjugated porphyrin oligomers. *J. Am. Chem. Soc.* **130**, 10171–10178 (2008).

11. Peeks, M. D., Neuhaus, P. & Anderson, H. L. Experimental and computational evaluation of the barrier to torsional rotation in a butadiyne-linked porphyrin dimer. *Phys. Chem. Chem. Phys.* **18**, 5264–5274 (2016).

12. Winters, M. U. *et al.* Photophysics of a butadiyne-linked porphyrin dimer: Influence of conformational flexibility in the ground and first singlet excited state. *J. Phys. Chem. C* **111**, 7192–7199 (2007).

13. El-Bayoumi, M. A., Dalle, J. P. & O’Dwyer, F. Fluorescence Lifetimes of Molecules that Undergo Large Configurational Changes upon Excitation. *J. Am. Chem. Soc.* **92**, 3494–3495 (1970).

14. Fenwick, O. *et al.* Linear and cyclic porphyrin hexamers as near infra-red emitters in organic light-emitting diodes. *Nano Lett.* **11**, 2451–2456 (2011).

15. List, E. J. W., Guentner, R., Scanducci de Freitas, P. & Scherf, U. The Effect of Keto Defect Sites on the Emission Properties of Polyfluorene-Type Materials. *Adv. Mater.* **14**, 374–378 (2002).

16. Yong, C.-K. *et al.* Ultrafast delocalization of excitation in synthetic light-harvesting nanorings. *Chem. Sci.* **6**, 181–189 (2015).

17. Hoffmann, M. *et al.* Enhanced π conjugation around a porphyrin[6] nanoring. *Angew. Chemie - Int. Ed.* **47**, 4993–4996 (2008).

18. Dey, A., Rao, A. & Kabra, D. A Complete Quantitative Analysis of Spatio-Temporal Dynamics of Excitons in Functional Organic Light-Emitting Diodes. *Adv. Opt. Mater.* **5**, 1600678 (2017).

19. Wallikewitz, B. H., Kabra, D., Gélinas, S. & Friend, R. H. Triplet dynamics in fluorescent polymer light-emitting diodes. *Phys. Rev. B - Condens. Matter Mater. Phys.* **85**, 22–25 (2012).

20. Murawski, C., Leo, K. & Gather, M. C. Efficiency roll-off in organic light-emitting diodes. *Adv. Mater.* **25**, 6801–6827 (2013).

21. Kim, J. *et al.* Molecular-scale interface engineering for polymer light-emitting diodes. *Nature* 481–484 (2000).

22. Kondakov, D. Y., Pawlik, T. D., Hatwar, T. K. & Spindler, J. P. Triplet annihilation exceeding spin statistical limit in highly efficient fluorescent organic light-emitting diodes. *J. Appl. Phys.* **106**, 124510 (2009).

23. Kim, J.-S., Ho, P. K. H., Greenham, N. C. & Friend, R. H. Electroluminescence emission pattern of organic light-emitting diodes: Implications for device efficiency calculations. *J. Appl. Phys.* **88**, 1073–1081 (2000).
